# Host Resistance, Population Structure and the Long-Term Persistence of Bubonic Plague: Contributions of a Modelling Approach in the Malagasy Focus

**DOI:** 10.1371/journal.pcbi.1003039

**Published:** 2013-05-09

**Authors:** Fanny Gascuel, Marc Choisy, Jean-Marc Duplantier, Florence Débarre, Carine Brouat

**Affiliations:** 1 IRD, CBGP (UMR IRD/INRA/CIRAD/MontpellierSupAgro), Montferrier-sur-Lez, France; 2 Ecole Normale Supérieure de Lyon, Lyon, France; 3 IRD, UMR 224, Montpellier, France; 4 Oxford University Clinical Research Unit-Hanoi, Hanoi, Vietnam; 5 CNRS, UMR 5554 ISEM, Montpellier, France; 6 Department of Zoology, University of British Columbia, Vancouver, Canada; 7 University of Idaho, Moscow, Idaho, United States of America; Pennsylvania State University, United States of America

## Abstract

Although bubonic plague is an endemic zoonosis in many countries around the world, the factors responsible for the persistence of this highly virulent disease remain poorly known. Classically, the endemic persistence of plague is suspected to be due to the coexistence of plague resistant and plague susceptible rodents in natural foci, and/or to a metapopulation structure of reservoirs. Here, we test separately the effect of each of these factors on the long-term persistence of plague. We analyse the dynamics and equilibria of a model of plague propagation, consistent with plague ecology in Madagascar, a major focus where this disease is endemic since the 1920s in central highlands. By combining deterministic and stochastic analyses of this model, and including sensitivity analyses, we show that (i) endemicity is favoured by intermediate host population sizes, (ii) in large host populations, the presence of resistant rats is sufficient to explain long-term persistence of plague, and (iii) the metapopulation structure of susceptible host populations alone can also account for plague endemicity, thanks to both subdivision and the subsequent reduction in the size of subpopulations, and extinction-recolonization dynamics of the disease. In the light of these results, we suggest scenarios to explain the localized presence of plague in Madagascar.

## Author Summary

Bubonic plague, known to have marked human history by three deadly pandemics, is an infectious disease which mainly circulates in wild rodent populations and is transmitted by fleas. Although this disease can be quickly lethal to its host, it has persisted on long-term in many rodent populations around the world. The reasons for this persistence remain poorly known. Two mechanisms have been invoked, but not yet explicitly and independently tested: first, the spatial structure of rodent populations (subdivision into several subpopulations) and secondly, the presence of, not only plague-susceptible rodents, but also plague-resistant ones. To gain insight into the role of the above two factors in plague persistence, we analysed a mathematical model of plague propagation. We applied our analyses to the case of Madagascar, where plague has persisted on central highlands since the 1920s and is responsible for about 30% of the human cases worldwide. We found that the long-term persistence of plague can be explained by the presence of any of the above two factors. These results allowed us to propose scenarios to explain the localized presence of plague in the Malagasy highlands, and help understand the persistence of plague in many wild foci.

## Introduction

Although bubonic plague has marked human history by three pandemics (Justinian plague in the 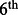
–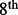 centuries, Medieval plague in the 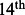
–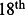 centuries and Asiatic plague since 1894 [1]), this zoonosis caused by the coccobacillus *Yersinia pestis* is primarily a rodent disease. Its persistent circulation in wild reservoirs is responsible for occasional epidemics in human populations [2], [3]. Each plague focus has distinct characteristics, but all have mammal hosts as reservoirs and fleas as vectors.

Two main factors are suspected to explain the endemic persistence of plague despite its high virulence. The first one is the coexistence of plague resistant and plague susceptible rodents in many wild foci of the world. Susceptible hosts are assumed to allow plague transmission by developing the high septicemia needed for the disease to spread, while resistant hosts would help maintain the host and flea populations and would lower the effective rate of encounter between infectious fleas and susceptible hosts [4]–[6]. The second factor which could explain plague endemism is the host metapopulation structure [7]–[10], which may allow for extinction-recolonization dynamics of plague in local foci, between which the disease spreads slowly [11], [12]. Theoretical models have shown that these extinction-recolonization dynamics are involved in the persistence of various infectious diseases, e.g. measles [8], [13], [14]. A few other mechanisms are thought to favour the persistence of plague, such as the presence of multiple hosts [5], [15], the possible persistence of *Y. pestis* in soils [16], [17], the direct transmission between rats inside burrows [18], [19] or the heterogeneity in the phenology of the host reproduction [15]. These alternative explanations will be discussed at the end of the article.

Explaining the endemism of plague has been the objective of several theoretical studies [15], [20]–[22]. However, the roles of resistance and of metapopulation structure of rodent reservoirs for plague persistence have rarely been explored separately. For instance, the model developed by Keeling & Gilligan [21] showed that metapopulation structure can explain the long-term persistence of plague via extinction-recolonization dynamics of the disease, but the theoretical population that they modelled included some resistant individuals, so that the roles of both factors cannot be disentangled. This is also the case for theoretical studies on the Kazakh focus [22], [23], where the hosts are modelled as partly resistant. In populations of susceptible hosts, such as prairie dogs (*Cynomys spp.*) in the United States, the link between spatial structure and plague persistence has been empirically observed [24]–[26] and theoretically confirmed, at least on short time scales (Salkeld et al. found that plague transmission between adjacent coteries of a structured susceptible host population lead to enzootic phases which last for more than 1 year in about 25% of model runs [27]). Also, several studies pointed out the need to test for the different mechanisms involved in plague endemism [5], [28].

Madagascar is one of the major plague foci in the world, accounting for 31% of the 50,000 reported human cases worldwide between 1987 and 2009 [29]. Bubonic plague was introduced in Madagascar in 1898 [30] and spread to central highlands in the 1920s [31]. Since that time, the disease persists in this region at the landscape level. In coastal areas and regions below 800 m of altitude, only sporadic urban epidemics occurred, due to human-mediated translocation of infected rodents from central highlands [30], [32]. In Madagascar, the main host of plague is the black rat, *Rattus rattus* [30], that is widespread throughout the island [30], while two species of fleas are involved as vectors [31]: *Xenopsylla cheopis*, the oriental rat flea, which has a cosmopolitan distribution, and *Synopsyllus fonquerniei*, an endemic flea from Madagascar whose distribution is restricted to central highlands. Compared to many other natural plague foci, only a few species are involved in the transmission of the disease [3]. Nevertheless, and despite its importance regarding public health, the causes of plague persistence in Madagascar have never been explored. Preliminary population genetic studies suggested that rat populations from the highlands are more geographically structured than those of the coastal areas, probably because of the more rugged physical landscape that limits migration [33]. Also, consistent with the hypothesis of a causal relationship between plague persistence and host resistance, at least 50% of the rats caught in Malagasy highlands are plague-resistant, whereas they are all susceptible in low altitude plague-free areas [34], [35]. However, as highlands were colonised by rats from coastal areas several centuries ago [34], the evolution of plague resistance in Malagasy black rats may be recent and posterior to the spread of the disease. It might thus be a consequence rather than a primary cause of plague persistence in rural areas of central highlands.

Building on the model of Keeling & Gilligan [20], [21], we developed a theoretical approach to evaluate independently the roles of host population structure and of host resistance in the long-term persistence of plague. The model was parameterized using data from plague ecology in Madagascar when they are available or from the literature otherwise. The sensitivity of the model to a range of parameters was tested. We evaluated the consistency of the hypothesis suggesting a recent evolution of plague resistance in Madagascar, and identified the parameters that need to be measured in order to test it.

## Materials and Methods

### Model

Our model of plague epidemiological dynamics is built on the framework developed by Keeling and Gilligan [20], [21] and is parameterized using data from studies on plague ecology in Madagascar when available [18], [34]–[36]. The system of differential equations (1) accounts for the number of individuals and epidemiological status of the rat (host) and fleas (vector) populations. The rodent host population is composed of three phenotypes: healthy, plague susceptible rats (whose number is 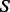 in system (1)), healthy, plague resistant rats (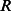), and infectious rats (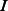). Two categories of vectors are taken into account: the mean number of fleas living on a rat (pulicidian index, 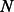) and the number of free infectious fleas (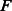).

The birth rate of rats is assumed to be density dependent [37] and modelled by a logistic equation, 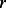 being the maximal birth rate and 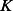 the carrying capacity of the rat population. The rats are assumed to die naturally at constant rate 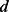. We assume no direct cost to resistance, however only a proportion 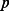 of the offspring of resistant rats are resistant (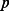 is the heritability of resistance; 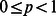), the other offspring (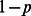) being all susceptible to plague [36]. In contrast, all the offspring of susceptible rats are born susceptible to plague [36]. Susceptible rats (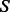) can contract the disease and become infectious (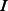) while resistant rats (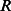) always remain uninfected, which is a realistic assumption in the context of the Malagasy plague [36]. Infection happens when free infectious fleas (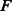) land on susceptible rats (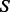) and transmit the bacillus according to the transmission parameter 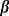. Free infectious fleas (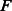) come randomly in contact with rats with a probability of encounter 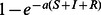 [38]. The parameter 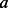 measures the search efficiency of fleas. Following [39], the infection of rats by fleas is modelled as a frequency-dependent process. We thus consider that the force of infection is 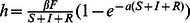. Infectious rats quickly die from septicemia, which results in an additional mortality term 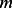, also called the virulence of the bacillus. The death of each of these rats leads to the release of 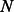 fleas in the environment, increasing the number of free infectious fleas (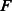). Free infectious fleas 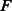 die at rate 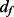. Fleas on the rats are assumed to have a density-dependent growth, with maximal growth rate 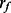 and a carrying capacity per rat 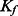. All these assumptions result in the following system of differential equations:
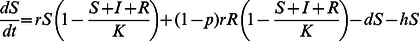
(1a)

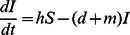
(1b)

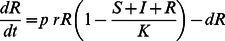
(1c)

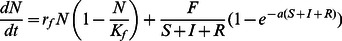
(1d)

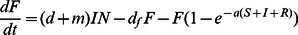
(1e)


**Figure 1:**
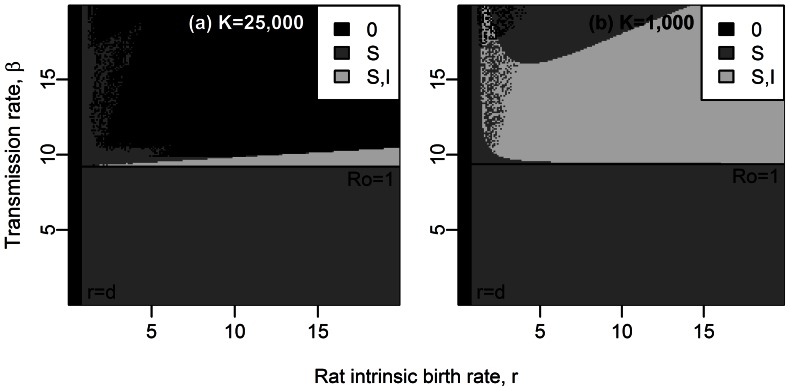
Equilibrium states for a susceptible population, according to the rats' maximal birth rate, 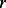, and the transmission rate, 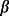, with (a) 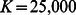 rats and (b) 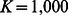 rats. Values for other parameters follow the ones presented in Table 1. Stable equilibrium states: (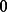,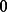) in black, (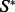,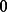) in dark grey and (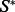,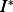) in light grey. The dynamics for four couples of parameters are given on Supporting Figure S4.

**Figure 2:**
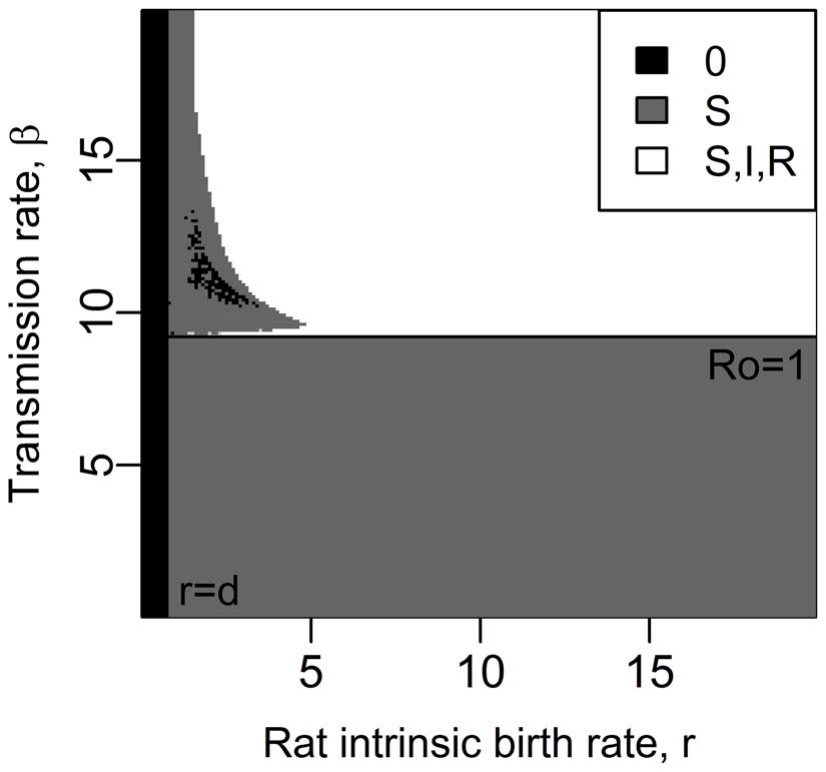
Equilibrium states for a rat population including resistant rats, according to the maximal birth rate of rats,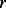, and the transmission rate, 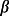. Parameter values are given in Table 1, 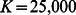 rats. Stable equilibrium states: (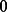,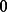,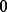) in black, (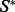,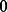,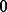) in dark grey, (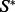,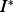,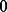) in light grey and (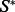,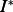,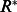) in white. The dynamics for four couples of parameters are given on Supporting Figure S8.

Our model includes several modifications compared to the model of Keeling & Gilligan [20], [21], in order to better depict wild plague foci, and specifically that of Madagascar. In our model, (i) infectious rats do not recover, as frequently observed [4], [6], [36], (ii) free infectious fleas either find a host or quickly die from starvation, a more explicit modelling of two events that were not distinguished in [21], and (iii) the descendants of resistant rats which are resistant also have a density-dependent birth rate, while they grew exponentially in [20]. Nevertheless, the above changes do not change the main characteristics of the outputs of the model (comparison of Figures 1 and 2 with Supporting Figures S1 and S2).

In the first steps of this study, model (1) is also analysed without the class of resistant rats (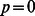 and no resistant rats initially in the system; see system (S1.1) in the Supporting Text S1), in order to investigate their role in plague persistence.

### Parameter values

The parameter values that we use come preferentially from experiments or field observations done in the context of the Malagasy plague focus. When relevant data are lacking, parameters are derived from values found in the plague literature (see Table 1).

**Table 1:** Parameter values and symbols used in this study, except if a different value is given in the legends.

	Parameter	Value	(Source)	From Madagascar
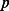	Heritability of resistance	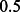	[35], [36]	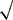
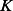	Carrying capacity of rats	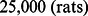	Arbitrary choice, 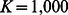 also studied	
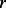	Maximal birth rate of rats	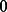 to 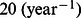	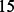 in [36], 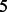 in [21]	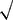
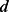	Natural mortality of rats	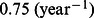	(J.-M Duplantier, unpublished data)	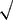
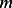	Additional mortality due to septicemia	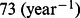	[34]	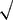
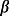	Transmission of the bacillus	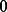 to 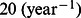	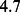 [21], 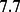 [49] and 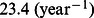 in [51]	
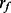	Growth of the fleas on rats	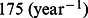	[68]	
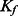	Carrying capacity of fleas per rat	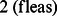	[18]	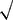
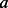	Search efficiency of free fleas	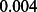	[21]	
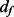	Natural mortality of free fleas	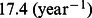	[6]	
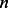	Number of subpopulations	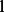 , 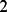 , 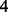 or 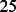	Arbitrary choices	
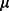	Proportion of inter-subpopulations infections	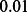	Rough estimate; 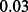 in [21]	

### Study of the equilibria

The basic reproductive number of a disease, 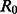, is the expected number of secondary cases caused by one infected individual introduced into a susceptible population [40]. A disease is expected to spread only if 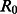 is greater than unity. We calculated 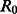 in our model using the Next Generation Approach [40], [41]. Details of the calculations are presented in the Supporting Text S2.

The system of differential equations (1) is then solved numerically, using the deSolve package [42] in R [43]. Deterministic simulations are run on a time long enough to ensure that equilibrium states are reached (typically 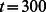 years with our parameters). When it is possible to find analytical solutions for the equilibria (for example with system (S1.2) without fleas, in the Supporting Text S1), we can check the accuracy of the numerical integrations of the model. There are four qualitative types of equilibrium states for the rat populations: (i) whole population extinction, labelled (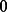, 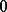) or (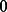, 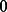, 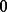) for the models without and with resistant rats, respectively; (ii) persistence of susceptible rats only, labelled (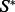, 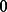) or (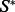, 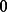, 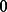); (iii) persistence of susceptible and infected rats but extinction of resistant rats (in systems containing resistant rats initially), (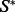, 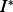) or (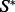, 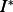, 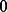); and finally (iv), in the model with resistant rats, coexistence of susceptible, infected and resistant rats, (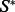, 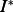, 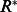). We consider a class to be extinct when the number of individuals drops at least once below 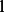 during the last 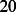 years of the numerical integration (to avoid any influence of the initial state of the system on the extinction criteria).

### Spatial structure: modelling metapopulations

To study the effect of spatial structure on disease persistence, a metapopulation of susceptible hosts only (i.e. without resistant hosts) of total carrying capacity equal to 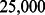 rats is modelled as a set of 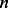 subpopulations of equal sizes. We neglect the effect of distance by assuming that all subpopulations are equidistant (a situation called island model [44] in population genetics). We consider (i) no spatial structure (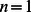), (ii) a weak spatial structure (ie, a low population subdivision) (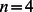) and (iii) a higher population subdivision (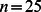). The fraction of infections that occur between subpopulations is given by the parameter 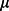. Although the rats in Madagascar may move temporarily to other subpopulations, thereby spreading the disease, capture-recapture studies have shown that these movements are temporary [18], [45]. We therefore model a migrating force of infection, instead of the migration of the rats themselves [46]. The value of 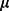 was estimated to be around 1% [18], [21].

The force of infection 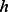 in system (1) thus becomes 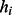 in the subpopulation 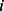, which includes the rats 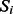, 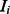 and the fleas 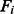:
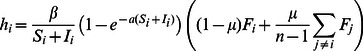
(2)


### Stochastic analyses

To assess the effect of population structure on the persistence of the disease, we use a stochastic version of our model without resistant rats (system (S1.1) in the Supporting Text S1), based on the Gillespie algorithm [47] that is implemented in the GillespieSSA R package [48]. It simulates a Markov stochastic process in continuous time and with discrete state values. We ran simulations both with and without metapopulation structure, in order to compare the persistence of plague (a hundred replications for each set of parameters). The number of simulations where susceptible rats 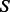 or infectious rats 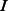 persist was recorded over time, to obtain an estimation of the probability of extinction of rats 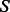 and 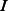 through time.

The scripts of the simulations (deterministic and stochastic) are deposited in the Dryad Repository: http://dx.doi.org/10.5061/dryad.55t60.

## Results

### Long-term plague persistence without resistance and without spatial structure

We first investigated the different outcomes of the model without resistance (variable 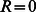) and without population structure (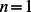), depending on the value of the transmission parameter 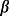 and of the maximal birth rate 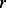.

When there are no resistant rats and no population structure (system (S1.1) in the Supporting Text S1), the rat population is viable if the maximal birth rate of rats 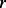 is larger than their mortality rate 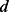 (Figures 1 and 2). The disease propagation threshold, 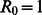, sets the limit between the rat population equilibria (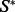,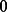) and (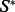,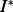). Using the Next Generation Method, we found
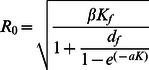
(3)


The propagation of plague is favoured by a high transmission from fleas to rats (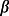), which increases the number of infectious rats, and by a high flea carrying capacity of rats (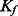), which increases the number of free fleas, the vectors of the disease (equation (3)). It is also favoured by a high carrying capacity of the rat population, 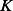, and by a high search efficiency of fleas, 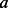, through their direct effect on the probability 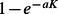 that a flea finds a host. Finally, a high mortality rate of free infectious fleas, 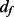, disadvantages disease propagation by limiting the number of vectors. However, note that the fact that plague can initially spread in a susceptible rat population, although necessary, is not a sufficient condition for the long-term persistence of the disease.

The parameters 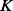 and 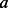 cannot for now be estimated from data collected in Madagascar, but the value of 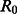 is not sensitive to changes in their values (see sensitivity analysis of 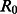 in Supporting Figure S3). The parameters 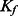 and 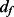 have more effect on the value of 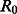 but their range of possible values is better known [6], [18]. As we did not have a precise estimate of the value of 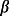 (the transmission rate varies between fleas and depends whether these are blocked or not; transmission efficiency was found to be about 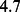 or 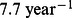 for blocked *X. cheopis* [49], [50], and 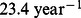 for unblocked *X. cheopis* [51]) and as it had a strong effect on the value of 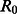, we investigated the outcomes of the model for a range of values of 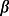 (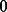 to 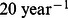) and calculated the critical transmission of the disease, 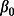, which is defined as the value of 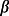 for 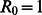 (see equation (4) below).

In the deterministic model, the disease initially spreads if and only if 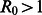, which is equivalent to
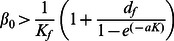
(4)


**Figure 3:**
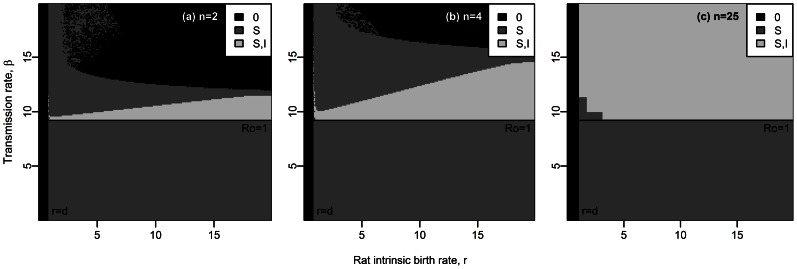
Equilibrium states for a susceptible host metapopulation composed of (a) 2 subpopulations, (b) 4 subpopulations and (c) 25 subpopulations (deterministic analysis). Total carrying capacity = 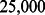 rats. Other parameter values are given in Table 1. Stable equilibrium states: (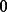,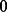) in black, (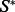,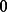) in dark grey and (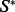,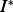) in light grey.

The threshold transmission parameter 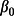 is plotted as a horizontal black line in Figures 1, 2 and 3; its values match with the values of 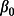 obtained by numerical simulations (Figure 1). However, as already mentioned, the 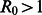 condition does not imply long-term persistence: Figure 1 shows that the equilibrium states with disease persistence (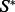,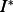) disappears when 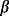 increases further above the critical transmission threshold 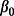, especially for large host populations. For 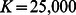 rats and values of 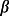 just above 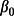, strong oscillations of the number of rats occur in each class, with low values between the peaks (Supporting Figures S4 and S5, 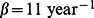). For higher values of 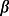, no oscillations happen but an epidemic wave decimates the host population (Supporting Figure S4, 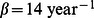): both the disease and the rat population therefore go extinct.

Without resistant rats, the disease can thus not persist in the long run within large host populations, except for a thin range of 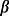 values. However, for smaller population sizes, the amplitude of the dynamics decreases (Supporting Figure S5), which prevents the extinction of the rat population and allows for disease persistence (Figure 1(b)). We thus observe that above the critical transmission, high host population sizes disadvantage the long-term persistence of plague.

It is worth noting that the same system without the flea compartment and with a direct disease transmission instead (system (S1.2) in the Supporting Text S1) shows stable equilibria when 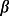 is above the critical transmission 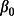 (Supporting Figure S6): the vectors therefore play a major role in the observed high amplitude dynamics leading to plague extinction by prolonging the infection process after the rats' death (free fleas infected by *Y. pestis* survive long enough to widely spread the disease). This point highlights the importance of accounting for the flea demography whenever studying the epidemiology of plague.

The above results are not highly dependent on the values of all other parameters linked to the behaviour of fleas and rats: changing these values may have a quantitative effect on the critical transmission 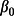 but it does not modify the qualitative behaviour of the system (see the sensitivity analysis of the equilibrium states on Supporting Figure S7).

### Long-term plague persistence with resistance, without spatial structure

When resistant rats 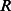 were included into the system (system (1)), three stable equilibrium states existed: (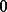,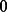,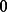), (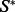,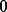,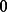), and (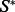,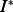,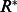) (Figure 2). The threshold 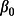 for disease propagation, i.e. 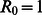, corresponds here to the limit between the equilibria (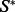,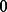,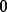) and (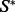,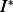,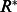). The expression of 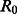 remains the same as without resistant hosts (see equation (3)).

Contrasting with the system without resistant rats, changing the carrying capacity 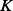 of the rat population does not influence the equilibrium states: when including resistant rats in the model, plague persists as long as 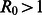 (Figure 2 and Supporting Figure S8). The sensitivity analysis showed that this result is not sensitive to changes in parameter values (Supporting Figure S9). The pattern of short epidemics followed by disease extinction that we previously observed and which was due to a lack of surviving susceptible rats, does not occur anymore because resistant rats allow the maintenance of not only resistant but also susceptible phenotypes (through partial heritability of resistance, 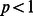) in the population.

### Long-term plague persistence with spatial structure, without resistance

In order to study the effect of spatial structure alone, we here assumed that resistant rats were absent (see system (S1.1) in the Supporting Text S1). The deterministic analysis of the system shows that host population structure alone allows for disease persistence when 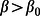 and 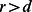 (Figure 3). Indeed, when the metapopulation is subdivided into enough subpopulations, oscillations in the number of healthy and infectious rats occur in each subpopulation, but the numbers of individuals stay above unity. Thus, host population structure allows plague persistence for parameter values where the disease would go to extinction in non-structured populations. Fragmentation turns large host populations which undergo high amplitude cycling dynamics (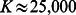 rats, Figures 1(a) and 3(a)) into small subpopulations which undergo dynamics of decreased amplitude (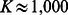 rats, Figures 1(b), 3(c) and Supporting Figure S5). However, if the total carrying capacity of the metapopulation is strongly decreased (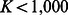 rats), then the densities of rats in each subpopulation become too low to allow for disease persistence.

**Figure 4:**
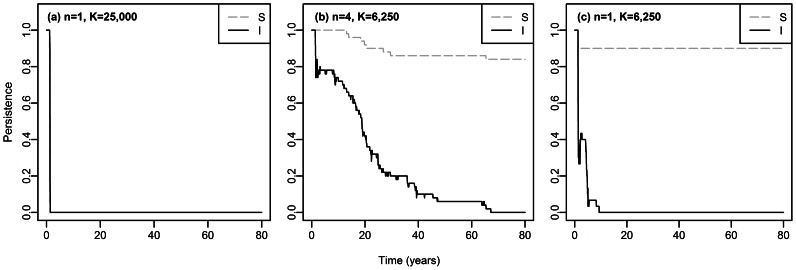
Estimated probability of persistence of susceptible rats 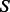 and infectious rats 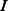 through time, in (a) one non structured population of 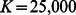 rats, (b) 4 subpopulations with total 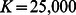 rats (ie, 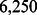 rats per subpopulation), and (c) one non structured population of 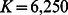 rats. This probability was estimated on 100 simulations. 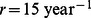, 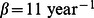 and other parameter values are given in Table 1. The Supporting Figure S10 illustrates, for one of the simulations in (b), the extinction-recolonization dynamics of the disease which occur between the subpopulations.

Stochastic analyses with parameter values such that 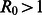 revealed that even a weak spatial structure increases the time of disease extinction by several decades (Figures 4(a) and 4(b)). The effect of population structure is twofold. First, population structure introduces extinction-recolonization dynamics of the disease between local foci (Supporting Figure S10), due to the asynchrony of the dynamics between subpopulations. Secondly, consistent with our deterministic results in non structured populations of susceptible rats, as long as 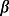 remains above the critical transmission 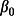 the disease persists for longer in smaller populations (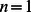 and 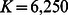 rats, no disease persistence after 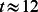 years across all our simulations; see Figure 4(c)) than in larger ones (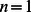 and 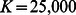 rats, no disease persistence after 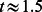 years in all our simulations; see Figure 4(a)). Thus, the longer persistence of the disease in the four-subpopulation metapopulation of 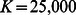 rats (no disease persistence after 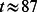 years; see Figure 4(b)) is due to both a population size reduction (in each subpopulation) and to extinction-recolonization dynamics of the disease. However, if population subdivision is too high, or the subpopulations too isolated (tested with 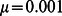 and 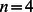), extinction time decreases again, as recolonization events become rare (Supporting Figure S11).

## Discussion

### Host population size and disease persistence

In rat populations without resistant rats, our results show that the persistence of the disease is favoured by intermediate population sizes. This may seem surprising given that the propagation of many infectious diseases is known to be favoured by larger host population sizes [52], [53]. However, disease invasion and persistence are two very different phenomenons [54], and the classically reported effect of population size on 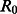 [53] is a matter of invasion rather than persistence. Here, the presence of vectors, the fleas, amplifies the spread of the disease (the fleas act as a very short-term external reservoir, [55], [56]) and thus triggers, after an intense epidemic, the extinction of the disease in large populations. Accordingly, other empirical studies reported that high host carrying capacities favour the invasion but not the persistence of plague. In Kazakhstan for instance, plague epidemics have been shown to be preceded by an increase in gerbil abundance over a minimum abundance threshold [22], [27], but the abundance of gerbils would predict plague endemicity (ie, long-term persistence) less than the probability of plague epidemics [23]. Heier et al. [23] suggested that if the initial spread of plague is faster when the population density of rodents is high, so is the extinction of the rodent population [23].

### Roles of resistance and structure

In large rat populations, we find that the presence of resistant rats alone may explain plague persistence. This confirms the hypothesis of a role for resistance in the endemism of plague [55]. Keeling & Gilligan [21] showed that if the initial proportion of resistant rats is below 20%, then short epizootics are more likely to occur than disease persistence. Previous theoretical studies [28], [57] assumed that resistant rats could play the role of plague reservoirs, by carrying infectious fleas, or that infectious rats could recover [21], whereby restoring the population of disease-sensitive rats. Our results show that these assumptions are not required to account for the persistence of this highly virulent disease, but that what matters most is the fact that resistant rats provide a source of new sensitive rats (since resistance is not totally heritable, 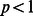).

Also, spatial structure alone may account for plague persistence. The possible recovery of infectious rats and the presence of resistant hosts were included in the model of Keeling and Gilligan [21], but we show here that they are not necessary to induce the long-term persistence of plague. A weak structure is enough to explain decades of disease persistence. It confirms what was already suggested by Salkeld et al. [27]. The effect of spatial structure is related to the combined effects of reduced subpopulation sizes and asynchrony between subpopulations. As in [58], [59], we indeed find that plague extinction takes longer for an intermediate force of coupling, 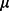, between subpopulations. Interestingly, the extinction-recolonization dynamics we observe happen to have about the same tempo as chronic re-emergences that have been recorded in some plague foci, such as the Kazakh focus, where epizootics last two to five years and occur every two to eight years [60]. Our results on the role of spatial structure are supported by field observations on prairie dogs (*Cynomys sp.*): in the United States, prairie dog colonies are on average smaller and separated by larger distances in regions where plague has historically been endemic than in regions where plague is historically absent [24]. Mortality due to *Y. pestis* is, for prairie dogs, close to 100%: the theoretical model we developed for susceptible rats may thus be applied to this example.

Both population subdivision alone and the presence of resistant rats may thus contribute to promote the persistence of plague in natural foci. However, the host population structure allows the persistence of the disease for a duration depending on the degree of spatial structure and on the features of the host and flea populations, while the presence of resistant hosts may allow a stable persistence of plague.

### Two hypotheses to explain the focal distribution of plague in Madagascar

In Madagascar, the plague focus is restricted to the central highlands. The focal persistence of plague may be explained by two different (non mutually exclusive) mechanisms, both of which will need to be validated through further field studies.

First, the differential persistence of plague may be due to different parameters in highlands and lowlands, such that 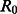 is above unity in the highlands and below unity in the lowlands. The few comparative studies that exist have not shown any major difference yet in biological parameter values related to rats between lowlands and highlands [3], [31] (J.-M. Duplantier, unpublished data). However, most of the parameter values linked to the fleas that we used have not been measured in Madagascar (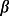, 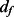, 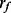, 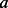). Some of these parameters (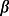, 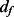) are among the ones which influence the basic reproductive number of the disease 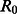 most. Climate is different in highlands and lowlands and may influence plague transmission by fleas [26], [61]. Moreover, flea communities are different, as one of the flea species (*S. fonquerniei*) is only in central highlands. The two flea species may not have the same demographic and transmission characteristics, and *S. fonquerniei* could play a role in the endemism of plague by being responsible for a high transmission: it has been shown to carry more bacillus *Y. pestis* during the plague season than *X. cheopis* [62]. Thus, further studies would be needed to experimentally compare the two flea species.

Even if both regions had 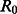 above unity (being or not equal), our results suggest that the persistence or extinction of the disease in each area may be explained by differences in the dynamics of the system, due to the presence/absence of resistant hosts and of population structure. In Madagascar, highlands were colonized by rats from Malagasy coastal populations some 800 years ago [63], long before the introduction of plague in the island. As no resistant phenotype, even at low frequency, has been found in rats from coastal populations [34], it seems likely that resistance evolved secondarily, after the spread of plague in highland rat populations. Population genetic studies showed that Malagasy rat populations are more genetically structured in landscapes characterised by sharp topographical relief, such as those found in some regions of the highlands, than in flat areas (Brouat et al., in revision). Rat population structure may thus have been more favourable to the persistence of the disease in the highlands than in coastal areas, and for periods of time sufficiently long to select resistance alleles. The evolution of resistance in the highlands may have in turn led to a more long-term plague persistence in this area. Host population structure and host resistance could thus have had a synergic effect to maintain plague in the Malagasy highlands. Testing this scenario would require more thorough theoretical studies based on the estimation of numerous biological parameters, especially in Malagasy flea populations (see above). Also, this requires the examination of the time needed for a resistance allele to invade a metapopulation depending on spatial structure. However, it is interesting to note that the scenario of a secondary evolution of host resistance in a restricted geographical range has already been identified for other diseases, such as malaria in Hawaii, for which resistance has only been selected in low altitude [64]. Also, an empirical analysis of the genetic structure of *Y. pestis* among prairie dogs in Arizona [25] suggests a dispersion dynamic of plague consistent with the above scenario. This latter study highlights the existence of two stages in plague propagation: first a phase of rapid expansion, through the encounter of a highly dense susceptible rodent population, and then a phase of decline of the host population and of extinction of the disease, except if slow and stable transmission cycles can arise either through resistant hosts, or spatially structured or low density susceptible populations [25].

### Conclusion

Using a simple model of plague propagation, we showed that both resistance and population subdivision may explain plague endemism. Madagascar may be a good illustration of how these two factors may act together, in synergy, to favour the long-term persistence of this highly virulent disease. However, further comparative field studies should aim at testing our assumptions on plague establishment in Madagascar, by trying to better assess the parameter values in the lowlands and highlands.

It is worth noting that several aspects of the cycle of plague transmission have been neglected in our study. Some of these could play an additional role in Madagascar, others should not have any impact, and all of them could be involved in plague persistence in other foci. Indeed, the existence of multi-plague reservoirs [5], [15], [55] seems unlikely in Madagascar, as *R. rattus* is margely dominant in rural communities, representing at least 95% of the captures [3], [65]. Also, although plague persistence in soils may exist in very peculiar situations (e.g., [66]) or in steppic environments [16], [17], it has never been demonstrated in Madagascar [17]. Alternatively, the heterogeneity in the phenology of the host reproduction [15], the direct transmission of *Y. pestis* inside burrows (through for example the release of the bacillus as aerosols [18], [19]), or the fact that resistant rats might be infectious for a short period of time before recovering (not shown for rats but observed for mice, [67]) or might release infectious fleas at their death could play additional roles in Madagascar and remain to be tested.

## Supporting Information

Figure S1
**Long-term plague persistence without resistant rats and without structure, based on Keeling & Gilligan's model.** Equilibrium states for a susceptible population, according to the rat's maximal birth rate, 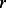, and the transmission rate, 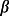, based on the model presented in [20]. (a) 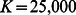 rats, (b) 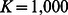 rats. Values for other parameters follow the ones presented in Table 1 and rats are assumed not to recover from plague infection (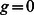 in Keeling & Gilligan's model). Stable equilibrium states: (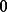,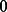) in black, (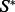,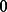) in dark grey and (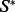,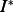) in light grey. The outcomes of the model developed by Keeling & Gilligan [20] are very similar to those obtained with our model simulated with the same parameter values (compare this figure with Figure 1 in the main text).(TIF)

Figure S2
**Long-term plague persistence with resistant rats and without structure, based on Keeling & Gilligan's model.** Equilibrium states for a rat population including resistant rats, according to the maximal birth rate of rats, 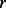, and the transmission rate, 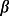, based on the model presented in [20]. Parameter values are given in Table 1, 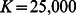 rats and rats are assumed not to recover from plague infection (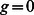 in Keeling & Gilligan's model). Stable equilibrium states: (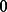,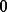,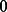) in black, (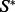,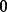,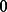) in dark grey, (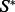,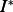,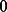) in light grey and (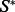,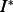,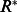) in white. The outcomes of the model developed by Keeling & Gilligan [20] are very similar to those obtained with our model simulated with the same parameter values (compare this figure with Figure 2 in the main text).(TIF)

Figure S3
**Sensitivity of the basic reproductive number of the disease,** 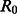 **, to parameter values,** for (a) 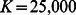 rats and (b) 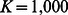 rats. The sensitivity corresponds to 
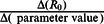
. It was calculated by increasing each paramater value by 10%.(TIF)

Figure S4
**Dynamics of the deterministic system without resistant rats (system (S1.1) in Supporting Text S1),** for 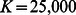 rats, 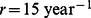 and (a) 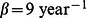, (b) 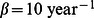, (c) 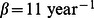 and (d) 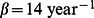. Values for other parameters follow the ones presented in Table 1. Time in years. Equilibria reached: (a) (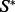,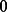), (b) (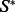,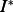), (c) (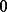,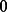) and (d) (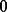,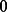).(TIF)

Figure S5
**Minimum and maximum values of the oscillations of the number of rats** 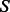 **and** 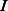 **(system (S1.1), without resistant rats, in the Supporting Text S1) according to the carrying capacity** 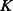 **,** for 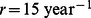 and (a) 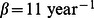, (b) 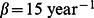. Values for other parameters follow the ones presented in Table 1. Only the numbers of rats 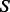 and 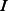 above 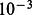 are shown (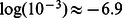).(TIF)

Figure S6
**Equilibrium states for susceptible rat populations in which the disease would spread without vectors (system (S1.2) in the Supporting Text S1).** 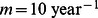. Values for other parameters follow the ones presented in Table 1. 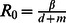. Stable equilibrium states: (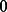,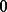) in black, (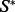,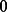) in dark grey and (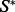,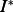) in light grey.(TIF)

Figure S7
**Sensitivity of the equilibrium states of the system without resistant rats (system (S1.1) in Supporting Text S1) to each parameter value,** given 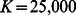 rats (a, c, e, g, i and k) and 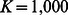 rats (b, d, f, h, j, l): parameter 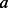 (a, b), 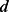 (c, d), 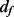 (e, f), 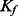 (g, h), 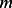 (i, j) and 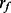 (k, l). Each parameter value is increased by 50% and other parameters values follow the ones presented in Table 1. Stable equilibrium states: (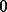,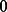) in black, (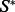,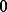) in dark grey and (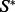,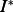) in light grey. This figure is to be compared with Figure 1.(TIF)

Figure S8
**Dynamics of the deterministic system with resistant rats (system (1)),** for 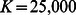 rats, 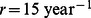 and for (a) 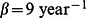 and (b) 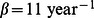. Values for other parameters follow the ones presented in Table 1. Time in years. Equilibria reached: (a) (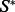,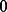,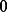) and (b) (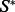,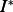,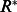).(TIF)

Figure S9
**Sensitivity of the equilibrium states of the system with resistant rats (system (0)) to each parameter value:** (a) parameter 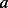, (b) 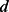, (c) 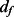, (d) 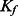, (e) 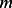, (f) 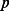, and (g) 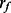. Each parameter value is increased by 50%, other parameters values follow the ones in Table 1, and 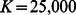 rats. Stable equilibrium states: (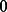,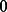,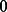) in black, (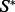,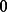,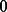) in dark grey, (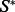,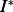,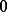) in light grey and (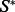,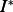,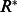) in white. This figure is to be compared with Figure 2.(TIF)

Figure S10
**Extinction-recolonisation dynamics.** Number of infectious rats 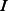 through time in each subpopulation, for one of the stochastic simulations performed in Figure 4(b). In blue: stochastic results, in orange: deterministic results. 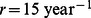, 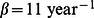 and other parameter values given in Table 1. The blue arrows indicate when the plague recolonizes a subpopulation from which it had disappeared (rescue effect). The disease never totally goes extinct in the deterministic model (epidemics still occur even if the number of infectious rats goes through extremely low values between the epidemics).(TIF)

Figure S11
**Effect of decreased force of coupling between subpopulations.** Estimated probability of persistence of susceptible rats 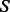 and infectious rats 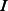 through time (measured on 60 simulations), for a metapopulation of 4 subpopulations with total 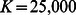 rats and with proportion of inter-subpopulation infections (force of coupling between subpopulations) 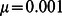. 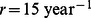, 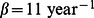 and other parameter values given in Table 1. This figure is to be compared with Figure 4(b).(TIF)

Text S1 **Other systems used.**
(PDF)

Text S2 **Calculation of the propagation threshold by the Next Generation Approach.**
(PDF)

## References

[1] Zietz B, Dunkelberg H (2004) The history of the plague and the research on the causative agent Yersinia pestis. Int J Hyg Envir Heal 207: 165–178. 10.1078/1438-4639-00259 PMC7128933 15031959

[2] Woolhouse M, Gowtage-Sequeria S (2005) Host range and emerging and reemerging pathogens. Emerg Infect Dis 11: 1842–1847. 16485468 10.3201/eid1112.050997 PMC3367654

[3] Duplantier JM, Duchemin JB, Chanteau S, Carniel E (2005) From the recent lessons of the Malagasy foci towards a global understanding of the factors involved in plague reemergence. Vet Res 36: 437–453. 15845233 10.1051/vetres:2005007

[4] Dennis D, Gage K, Gratz N, Poland J, Tikhomirov E (1999) Plague manual: epidemiology, distribution, surveillance and control. Geneva: World Health Organization.

[5] Gage K, Kosoy M (2005) Natural history of plague: perspectives from more than a century of research. Ann Rev Entomol 50: 505–528. 15471529 10.1146/annurev.ento.50.071803.130337

[6] Eisen R, Eisen L, Gage K (2009) Studies of vector competency and e_ciency of north American eas for *Yersinia pestis*: state of the field and future research needs. J Med Entomol 46: 737–744. 19645275 10.1603/033.046.0403

[7] Hanski I (1999) Metapopulation ecology. New York: Oxford University Press., 328 pp.

[8] Bolker B, Grenfell B (1995) Space, persistence and dynamics of measles epidemics. Proc R Soc B 348: 309–320. 10.1098/rstb.1995.0070 8577828

[9] Cully J, Williams E (2001) Interspecific comparisons of sylvatic plague in prairie dogs. J Mammal 82: 894–905.

[10] Davis S, Klassovskiy N, Ageyev V, Suleimenov B, Atshabar B, et al (2007) Plague metapopulation dynamics in a natural reservoir: the burrow system as the unit of study. Epidemiol Infect 135: 740–748. 17156497 10.1017/S095026880600759X PMC2870638

[11] Collinge S, Johnson W, Ray C, Matchett R, Grensten J, et al (2005) Landscape structure and plague occurrence in black-tailed prairie dogs on grasslands of the western USA. Landscape Ecol 20: 941–955.

[12] Stapp P, Salkeld D, Eisen R, Pappert R, Young J, et al (2008) Exposure of small rodents to plague during epizootics in black-tailed prairie dogs. J Wildl Dis 44: 724–730. 18689662 10.7589/0090-3558-44.3.724

[13] Hess G (1996) Disease in metapopulations models: implications for conservation. Ecology 77: 1617–1632.

[14] Hagenaars T, Donnelly C, Ferguson N (2004) Spatial heterogeneity and the persistence of infectious diseases. J Theor Biol 229: 349–359. 15234202 10.1016/j.jtbi.2004.04.002

[15] Foley P, Foley J (2010) Modeling susceptible infective recovered dynamics and plague persistence in California rodent-flea communities. Vector-borne Zoonotic Dis 10: 59–67. 20158333 10.1089/vbz.2009.0048

[16] Baltazard M (1964) The conservation of plague in inveterate foci. J Hyg Epidemiol Microbiol Immunol 120: 409–421. 14238939

[17] Mollaret H (1963) Conservation experimentale de la peste dans le sol. Bull Soc Pathol Exot 56: 11681182. 14156818

[18] Rahelinirina S, Duplantier JM, Ratovonjato J, Ramilijaona O, Ratsimba M, et al (2010) Study on the movement of *Rattus rattus* and evaluation of the plague dispersion in Madagascar. Vector-borne Zoonotic Dis 10: 77–84. 20158335 10.1089/vbz.2009.0019

[19] Agar S, Sha J, Foltz S, Erova T, Walberg K, et al (2009) Characterization of the rat pneumonic plague model: infection kinetics following aerosolization of *Yersinia pestis* co92. Microbes Infect 11: 205–214. 19073275 10.1016/j.micinf.2008.11.009

[20] Keeling M, Gilligan C (2000) Metapopulation dynamics of bubonic plague. Nature 407: 903–905. 11057668 10.1038/35038073

[21] Keeling M, Gilligan C (2000) Bubonic plague: a metapopulation model of a zoonosis. Proc R Soc B 267: 2219–2230. 10.1098/rspb.2000.1272 PMC1690796 11413636

[22] Davis S, Trapman P, Leirs H, Begon M, Heesterbeek JP (2008) The abundance threshold for plague as a critical percolation phenomenon. Nature 454: 634–637. 18668107 10.1038/nature07053

[23] Heier L, Storvik G, Davis S, Viljugrein H, Ageyev V, et al (2011) Emergence, spread, persistence and fade-out of sylvatic plague in Kazakhstan. Proc R Soc B 278: 2915–2923. 10.1098/rspb.2010.2614 PMC3151704 21345866

[24] Cully J, Johnson T, Collinge S, Ray C (2010) Disease limits populations: plague and black-tailed prairie dogs. Vector-borne Zoonotic Dis 10: 7–15. 20158327 10.1089/vbz.2009.0045 PMC2945311

[25] Girard J, Wagner D, Vogler A, Keys C, Allender C, et al (2004) Differential plague-transmission dynamics determine *Yersinia pestis* population genetic structure on local, regional, and global scales. Proc Natl Acad Sci USA 101: 8408–8413. 15173603 10.1073/pnas.0401561101 PMC420407

[26] Snäll T, O'Hara R, Ray C, Collinge S (2008) Climate-driven spatial dynamics of plague among prairie dog colonies. Am Nat 171: 238–248. 18197776 10.1086/525051

[27] Salkeld D, Salathé M, Stapp P, Jones J (2010) Plague outbreaks in prairie dog populations explained by percolation thresholds of alternate host abundance. Proc Natl Acad Sci USA 107: 14247–14250. 20660742 10.1073/pnas.1002826107 PMC2922574

[28] Foley J, Zipser J, Chomel B, Girvetz E, Foley P (2007) Modeling plague persistence in host-vector communities in California. J Wildl Dis 43: 408–424. 17699079 10.7589/0090-3558-43.3.408

[29] WHO (2010) Human plague: review of regional morbidity and mortality; 2004–2009. Weekly Epidemiological Record of the World Health Organization 85: 37–48.

[30] Chanteau S (2006) Atlas de la peste à Madagascar. Paris: IRD/Institut Pasteur/AUF Editions.

[31] Brygoo E (1966) Epidemiology of the plague at Madagascar. Med Trop 26: Suppl. 26–79. 6011187

[32] Vogler A, Chan F, Wagner D, Roumagnac P, Lee J, et al (2011) Phylogeography and molecular epidemiology of *Yersinia pestis* in Madagascar. PLoS Negl Trop Dis 5: e1319. 21931876 10.1371/journal.pntd.0001319 PMC3172189

[33] Gilabert A (2005) Génétique des populations de rats noirs (*Rattus rattus*) de Madagascar en relation avec la peste. Master's thesis, Paris: University Paris XI. 25 pp.

[34] Tollenaere C, Rahalison L, Ranjalahy M, Duplantier JM, Rahelinirina S, et al (2010) Susceptibility to *Yersinia pestis* experimental infection in wild Rattus rattus, reservoir of plague in Madagascar. EcoHealth 7: 242–247. 20443044 10.1007/s10393-010-0312-3

[35] Rahalison L, Ranjalahy M, Duplantier JM, Duchemin JB, Ravelosaona J, et al (2003) Susceptibility to plague of the rodents in Antananarivo, Madagascar. Adv Exp Med Biol 529: 439–442. 12756805 10.1007/0-306-48416-1_87

[36] Tollenaere C (2009) Génétique et évolution du rat noir, *Rattus rattus*, réservoir de la peste à Madagascar. [Ph.D. thesis] Montpellier: University Montpellier II. 208 p. URL http://www.mpl.ird.fr/ci/masto/infos/033b.pdf.

[37] Leirs H, Stenseth N, Nichols J, Hines J, Verhagens R, et al (1997) Stochastic seasonality and non linear density-dependent factors regulate population size in an African rodent. Nature 389: 176–180. 9296494 10.1038/38271

[38] Nicholson A, Bailey V (1935) The balance of animal populations. Proc Zool Soc Lond 105: 551–598.

[39] McCallum H, Barlow N, Hone J (2001) How should pathogen transmission be modelled? Trends Ecol Evol 16: 295–300. 11369107 10.1016/s0169-5347(01)02144-9

[40] Diekmann O, Heesterbeek J (2000) Mathematical epidemiology of infectious diseases: model building, analysis and interpretation. Chichester: John Wiley.

[41] Hurtford A, Cownden D, Day T (2010) Next-generation tools for evolutionary invasion analyses. J R Soc Interface 7: 561–571. 19955121 10.1098/rsif.2009.0448 PMC2842787

[42] Soetaert K, Petzoldt T, Woodrow Setzer R (2010) Solving Differential Equations in R: Package deSolve. Available: http://jstatsoft.org/v33/i09/.

[43] R Development Core Team (2011) R: A Language and Environment for Statistical Computing. Vienna (Austria): R Foundation for Statistical Computing. Available: http://www.r-project.org.

[44] Haldane J (1930) A mathematical theory of natural and artificial selection. VI. Isolation. Math Proc Camb Phil Soc 26: 220–230.

[45] Meehan A (1984) Rats and mice: their biology and control. East Grinstead: Rentokil Ltd, 383 pp.

[46] Medlock J, Kot M (2003) Spreading disease: Integro-differential equations old and new. Math Biosci 184: 201–222. 12832148 10.1016/s0025-5564(03)00041-5

[47] Gillespie D (1977) Exact stochastic simulation of coupled chemical reactions. J Phys Chem 81: 2340–2361.

[48] Pineda-Krch M (2010) GillespieSSA: a stochastic simulation package for R. Available: http://pineda-krch.com/gillespiessa.

[49] Lorange E, Race B, Sebbane F, Hinnebusch B (2005) Poor vector competence of eas and the evolution of hypervirulence in *Yersinia pestis* . J Infect Dis 191: 1907–1912. 15871125 10.1086/429931

[50] Macchiavello A (1954) Reservoirs and vectors of plague. J Trop Med Hyg 57: 1–68. 13131385

[51] Eisen R, Wilder A, Bearden S, Montenieri J, Gage K (2007) Early-phase transmission of *Yersinia pestis* by unblocked *Xenopsylla cheopis (Siphonaptera: Pulicidae)* is as efficient as transmission by blocked eas. J Med Entomol 44: 678–682. 17695025 10.1603/0022-2585(2007)44[678:etoypb]2.0.co;2

[52] Keeling M, Grenfell B (1997) Disease extinction and community size: modeling the persistence of measles. Science 275: 65–67. 8974392 10.1126/science.275.5296.65

[53] Anderson R, May R (1979) Population biology of infectious diseases. Part I Nature 280: 361–367. 10.1038/280361a0 460412

[54] Lloyd-Smith J, Cross P, Briggs C, Daugherty M, GetzW, et al (2005) Should we expect population thresholds for wildlife disease? Trends Ecol Evol 20: 511–519. 16701428 10.1016/j.tree.2005.07.004

[55] Wimsatt J, Biggins D (2009) A review of plague persistence with special emphasis on eas. Vectorborne Zoonotic Dis 46: 85–99. 19502688

[56] Buhnerkempe M, Eisen R, Goodell B, Gage K, Antolin M, et al (2011) Transmission shifts underlie variability in population responses to *Yersinia pestis* infection. PLoS One 6: e22498. 21799873 10.1371/journal.pone.0022498 PMC3143141

[57] Webb C, Brooks C, Gage K, Antolin M (2006) Classic ea-borne transmission does not drive plague epizootics in prairie dogs. Proc Natl Acad Sci USA 103: 6236–6241. 16603630 10.1073/pnas.0510090103 PMC1434514

[58] Jesse M, Ezanno P, Davis S, Heesterbeek J (2008) A fully coupled, mechanistic model for infectious disease dynamics in a metapopulation: movement and epidemic duration. J Theor Biol 254: 331–338. 18577388 10.1016/j.jtbi.2008.05.038

[59] Jesse M, Heesterbeek H (2011) Divide and conquer? Persistence of infectious agents in spatial metapopulations of hosts. J Theor Biol 275: 12–20. 21276802 10.1016/j.jtbi.2011.01.032

[60] Davis S, Begon M, Bruyn LD, Ageyev V, Klassovskiy N, et al (2004) Predictive thresholds for plague in Kazakhstan. Science 304: 736–738. 15118163 10.1126/science.1095854

[61] Cavanaugh D (1971) Specific effect of temperature upon transmission of the plague bacillus by the oriental rat flea, *Xenopsylla Cheopis* . Am J Trop Med Hyg 20: 264–273. 5553266 10.4269/ajtmh.1971.20.264

[62] Rahelinirina S (2009) Risque pesteux dans les foyers ruraux du Moyen Ouest malgache: déplacements et structuration des populations de rats noirs *Rattus rattus* (Linnaeus, 1758) de l'échelle de l'habitat à celle du paysage. Ph.D. thesis, Antananarivo: University of Antananarivo. 128 pp.

[63] Tollenaere C, Brouat C, Duplantier JM, Rahalison L, Rahelinirina S, et al (2010) Phylogeography of the introduced species *Rattus rattus* in the western Indian Ocean, with special emphasis on the colonization history of Madagascar. J Biogeogr 37: 398–410.

[64] Anderson T (2009) Mapping the spread of malaria drug resistance. PLoS Med 6: e1000054. 19365538 10.1371/journal.pmed.1000054 PMC2661254

[65] Duplantier J, Rakotondravony D (1999) The rodent problem in Madagascar : agricultural pest and threat to human health. In: H. L. Singleton G, Leirs H and Zhang Z, editors. Ecologically-based rodent management. ACIAR publ., 441–459 pp.

[66] Eisen R, Petersen J, Higgins C, Wong D, Levy C, et al (2008) Persistence of *Yersinia pestis* in soil under natural conditions. Emerg Infect Dis 14: 941–943. 18507908 10.3201/eid1406.080029 PMC2600287

[67] Demeure C, Blanchet C, Fitting C, Fayolle C, Khun H, et al (2011) Early systemic bacterial dissemination and rapid innate immune response characterize genetic resistance to plague of seg mice. J Infect Dis 205: 134–143. 22090450 10.1093/infdis/jir696

[68] Pattons W, Evans A (1929) Insects, ticks, mites and venomous animals of medical and veterinary importance. Part I-Medical. Croydon: H. R. Grubb Ltd., 785 pp.

