# Supplementary figures and images for: Host Resistance, Population Structure and the Long-Term Persistence of Bubonic Plague: Contributions of a Modelling Approach in the Malagasy Focus
Source: PLoS Comput Biol. 2013 May 9;9(5):e1003039. doi: 10.1371/journal.pcbi.1003039 (PMC3649974; doi:10.1371/journal.pcbi.1003039)

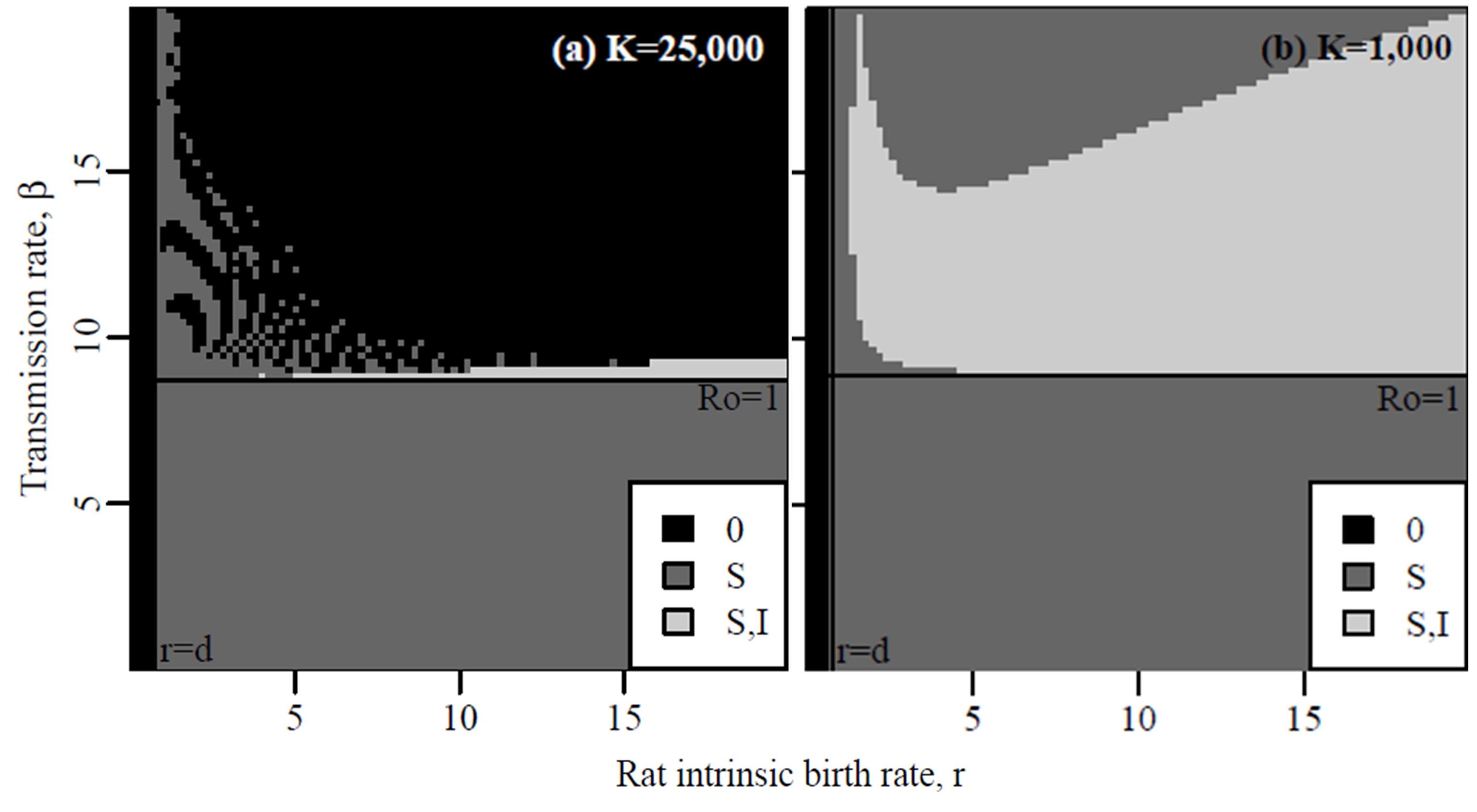

Supplement: Figure S1 — Long-term plague persistence without resistant rats and without structure, based on Keeling & Gilligan's model. Equilibrium states for a susceptible population, according to the rat's maximal birth rate, , and the transmission rate, , based on the model presented in [20]. (a) rats, (b) rats. Values for other parameters follow the ones presented in Table 1 and rats are assumed not to recover from plague infection ( in Keeling & Gilligan's model). Stable equilibrium states: (,) in black, (,) in dark grey and (,) in light grey. The outcomes of the model developed by Keeling & Gilligan [20] are very similar to those obtained with our model simulated with the same parameter values (compare this figure with Figure 1 in the main text). (TIF) [file pcbi.1003039.s001.tif]

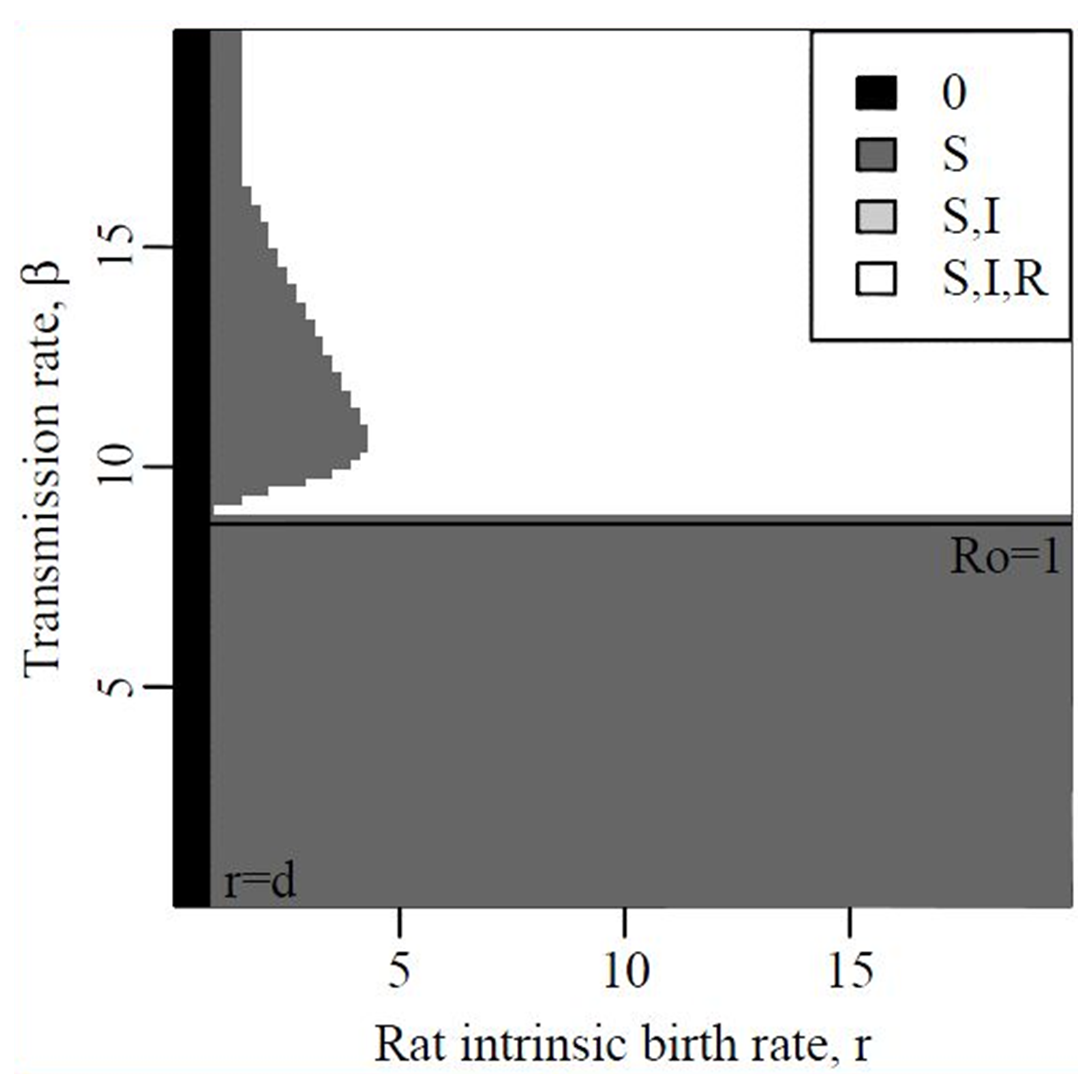

Supplement: Figure S2 — Long-term plague persistence with resistant rats and without structure, based on Keeling & Gilligan's model. Equilibrium states for a rat population including resistant rats, according to the maximal birth rate of rats, , and the transmission rate, , based on the model presented in [20]. Parameter values are given in Table 1, rats and rats are assumed not to recover from plague infection ( in Keeling & Gilligan's model). Stable equilibrium states: (,,) in black, (,,) in dark grey, (,,) in light grey and (,,) in white. The outcomes of the model developed by Keeling & Gilligan [20] are very similar to those obtained with our model simulated with the same parameter values (compare this figure with Figure 2 in the main text). (TIF) [file pcbi.1003039.s002.tif]

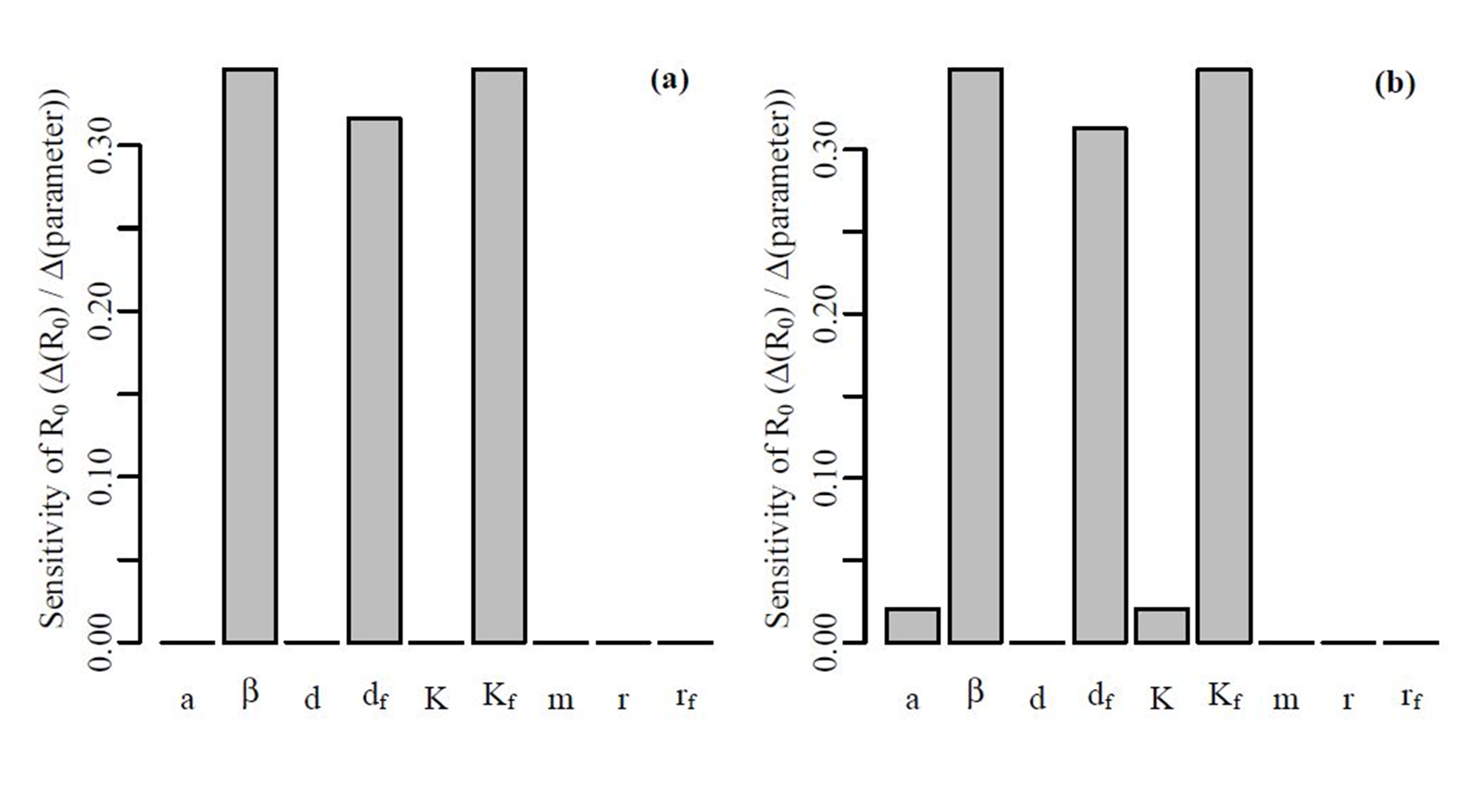

Supplement: Figure S3 — Sensitivity of the basic reproductive number of the disease, , to parameter values, for (a) rats and (b) rats. The sensitivity corresponds to . It was calculated by increasing each paramater value by 10%. (TIF) [file pcbi.1003039.s003.tif]

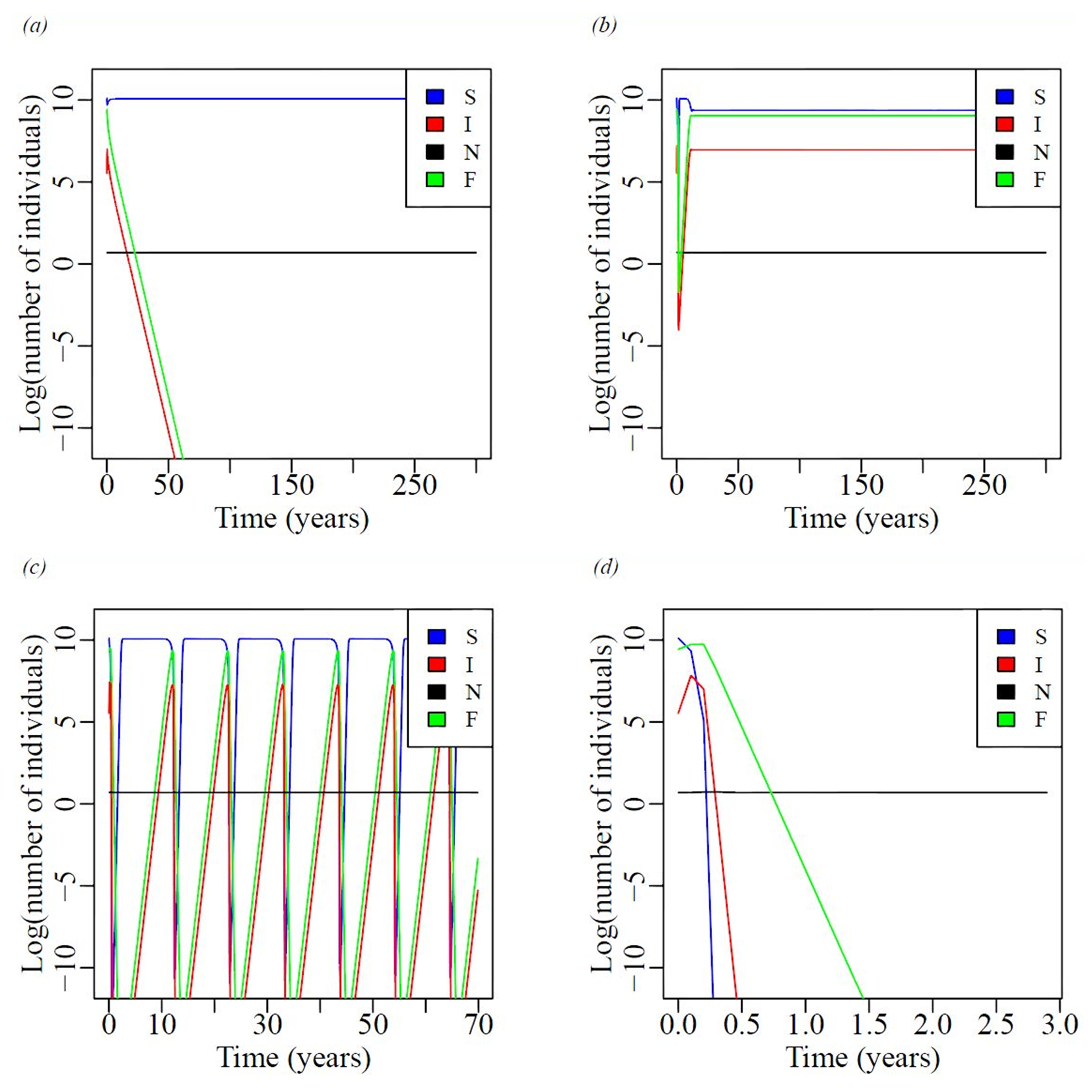

Supplement: Figure S4 — Dynamics of the deterministic system without resistant rats (system (S1.1) in Supporting Text S1), for rats, and (a) , (b) , (c) and (d) . Values for other parameters follow the ones presented in Table 1. Time in years. Equilibria reached: (a) (,), (b) (,), (c) (,) and (d) (,). (TIF) [file pcbi.1003039.s004.tif]

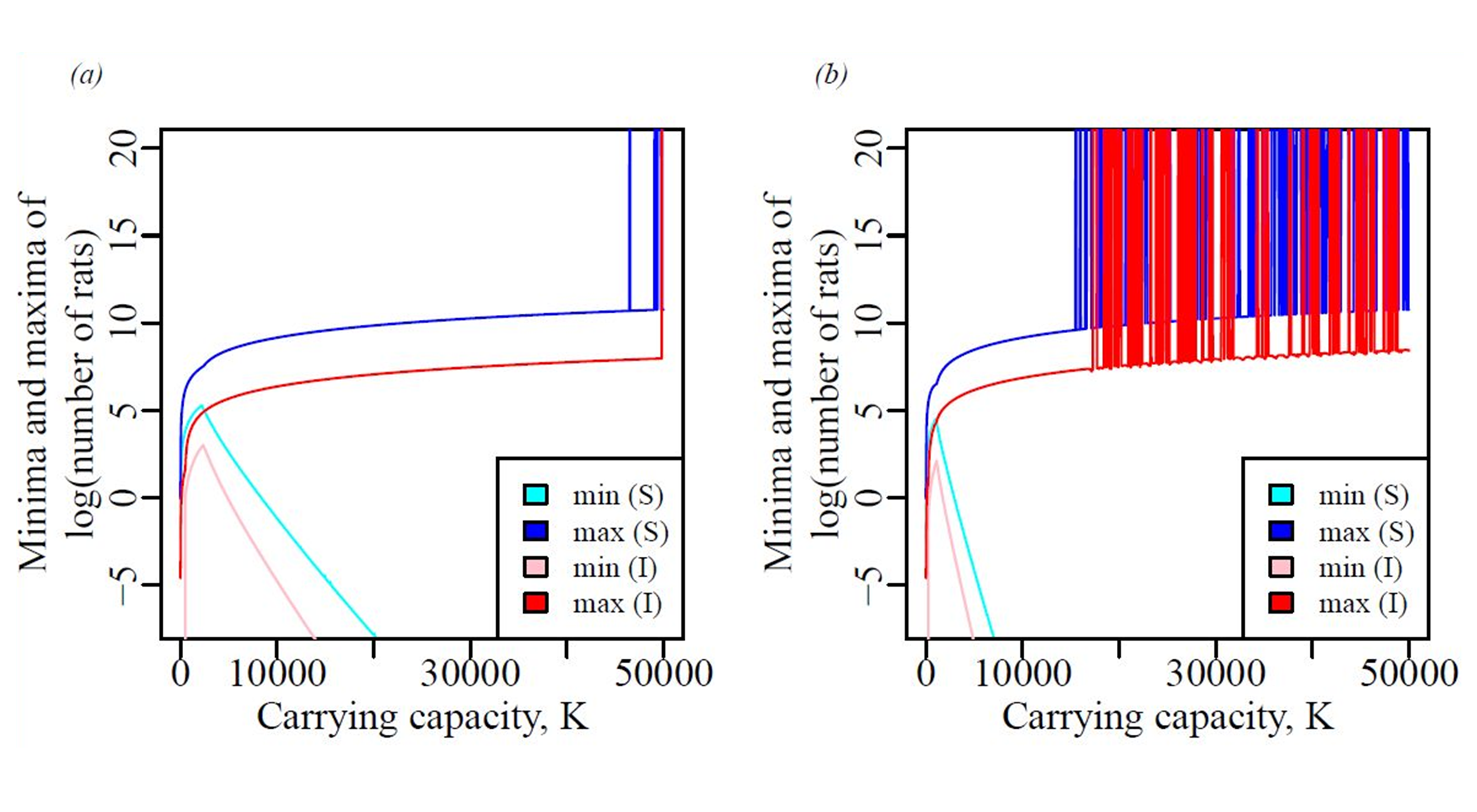

Supplement: Figure S5 — Minimum and maximum values of the oscillations of the number of rats and (system (S1.1), without resistant rats, in the Supporting Text S1) according to the carrying capacity , for and (a) , (b) . Values for other parameters follow the ones presented in Table 1. Only the numbers of rats and above are shown (). (TIF) [file pcbi.1003039.s005.tif]

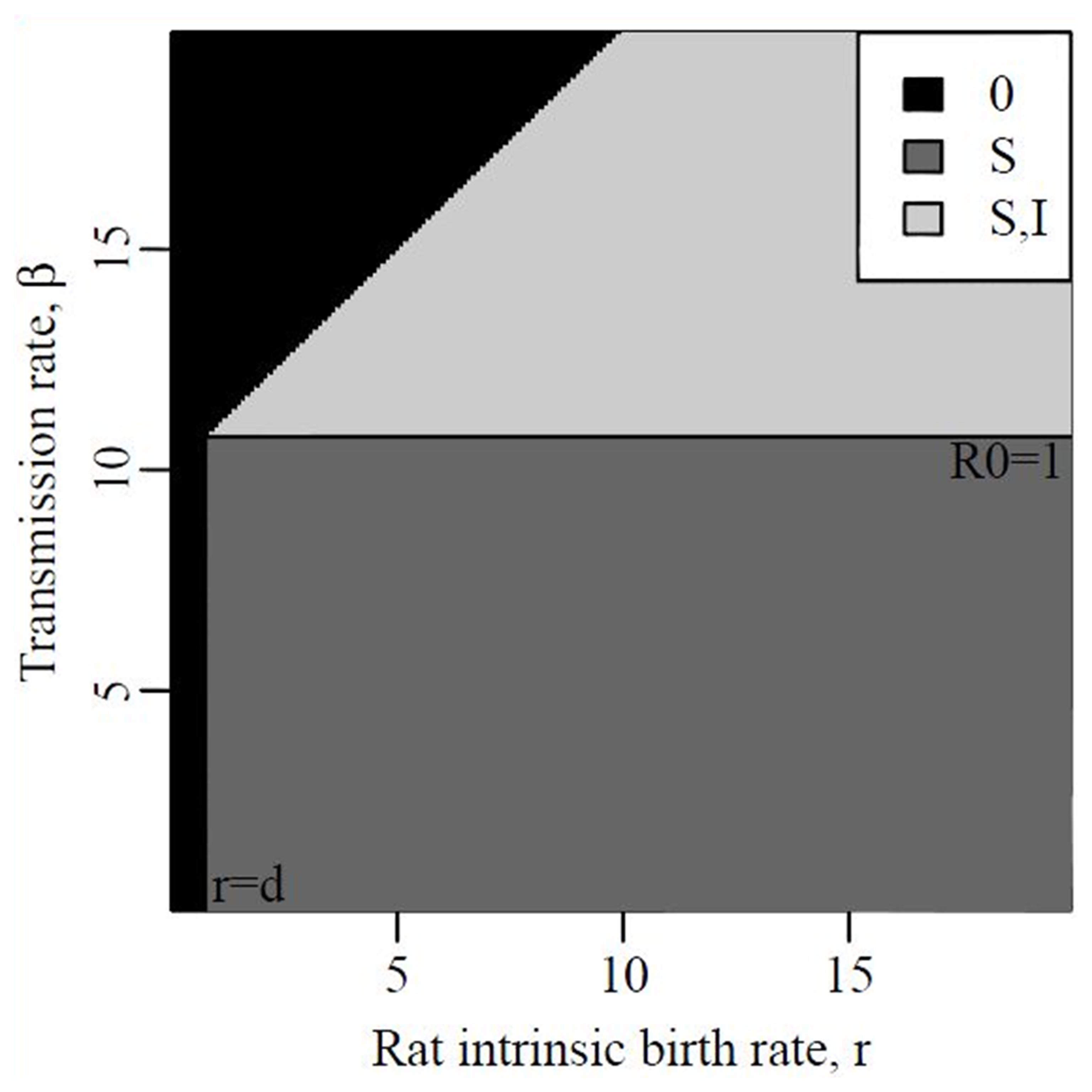

Supplement: Figure S6 — Equilibrium states for susceptible rat populations in which the disease would spread without vectors (system (S1.2) in the Supporting Text S1). . Values for other parameters follow the ones presented in Table 1. . Stable equilibrium states: (,) in black, (,) in dark grey and (,) in light grey. (TIF) [file pcbi.1003039.s006.tif]

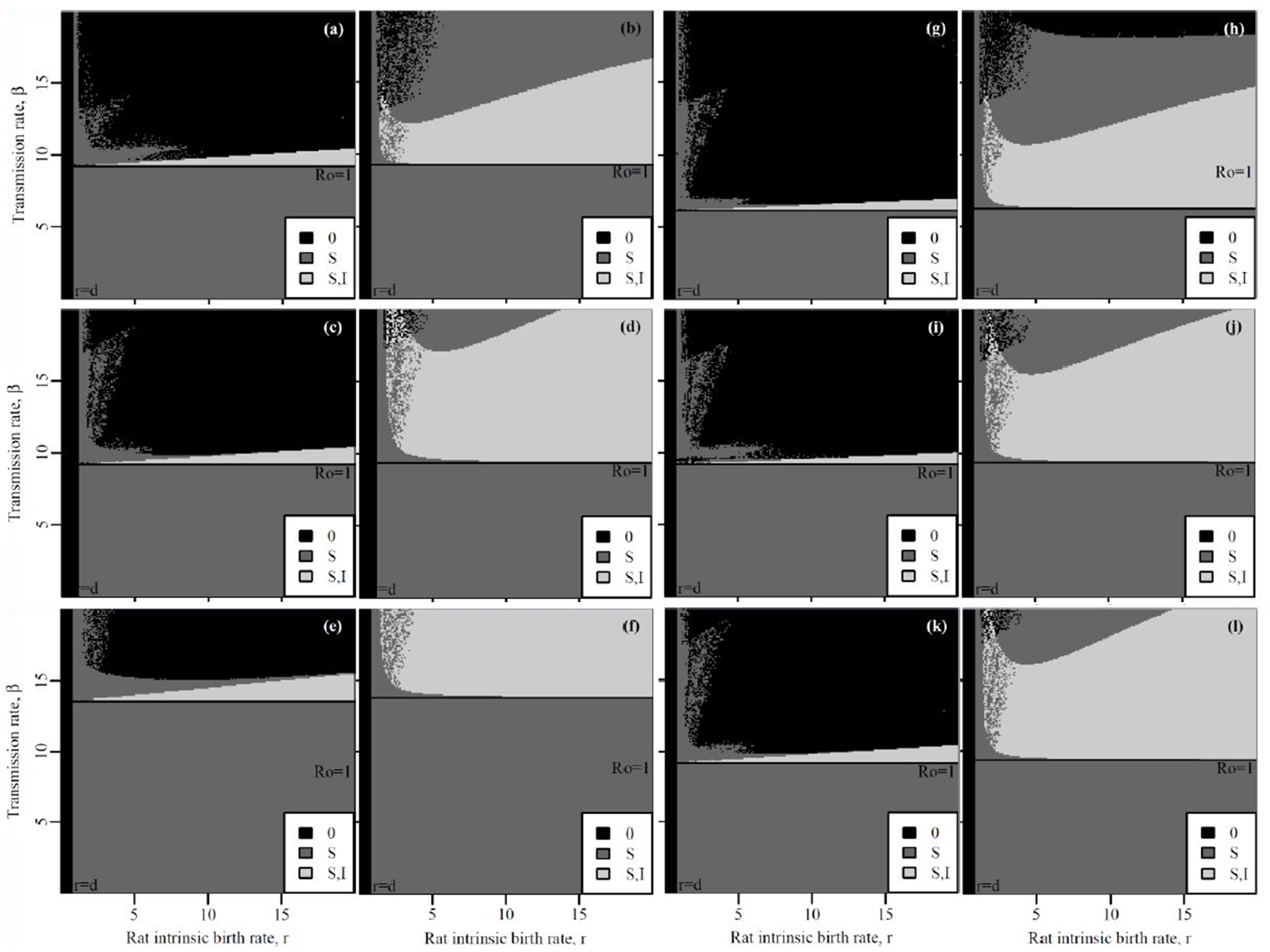

Supplement: Figure S7 — Sensitivity of the equilibrium states of the system without resistant rats (system (S1.1) in Supporting Text S1) to each parameter value, given rats (a, c, e, g, i and k) and rats (b, d, f, h, j, l): parameter (a, b), (c, d), (e, f), (g, h), (i, j) and (k, l). Each parameter value is increased by 50% and other parameters values follow the ones presented in Table 1. Stable equilibrium states: (,) in black, (,) in dark grey and (,) in light grey. This figure is to be compared with Figure 1. (TIF) [file pcbi.1003039.s007.tif]

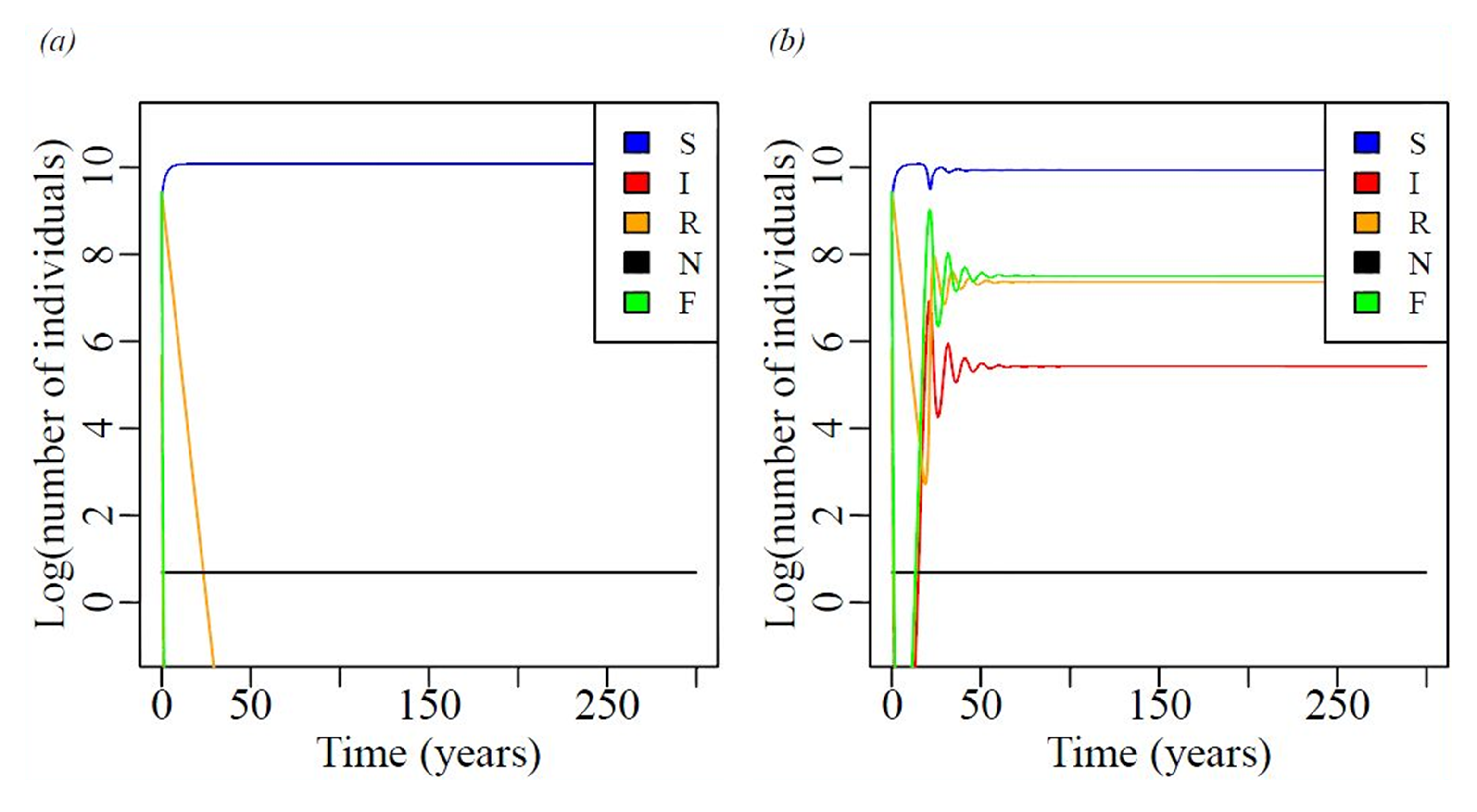

Supplement: Figure S8 — Dynamics of the deterministic system with resistant rats (system (1)), for rats, and for (a) and (b) . Values for other parameters follow the ones presented in Table 1. Time in years. Equilibria reached: (a) (,,) and (b) (,,). (TIF) [file pcbi.1003039.s008.tif]

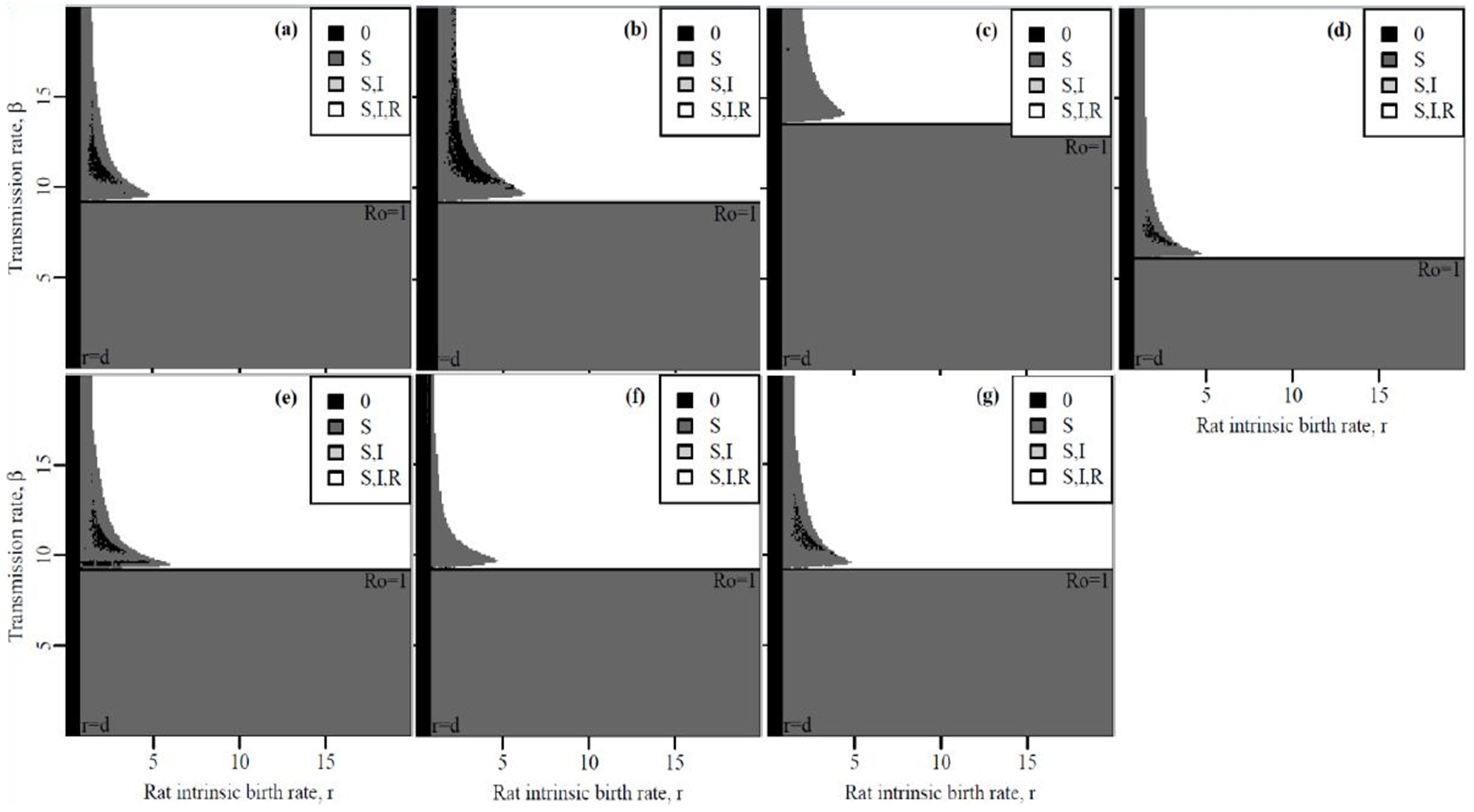

Supplement: Figure S9 — Sensitivity of the equilibrium states of the system with resistant rats (system (0)) to each parameter value: (a) parameter , (b) , (c) , (d) , (e) , (f) , and (g) . Each parameter value is increased by 50%, other parameters values follow the ones in Table 1, and rats. Stable equilibrium states: (,,) in black, (,,) in dark grey, (,,) in light grey and (,,) in white. This figure is to be compared with Figure 2. (TIF) [file pcbi.1003039.s009.tif]

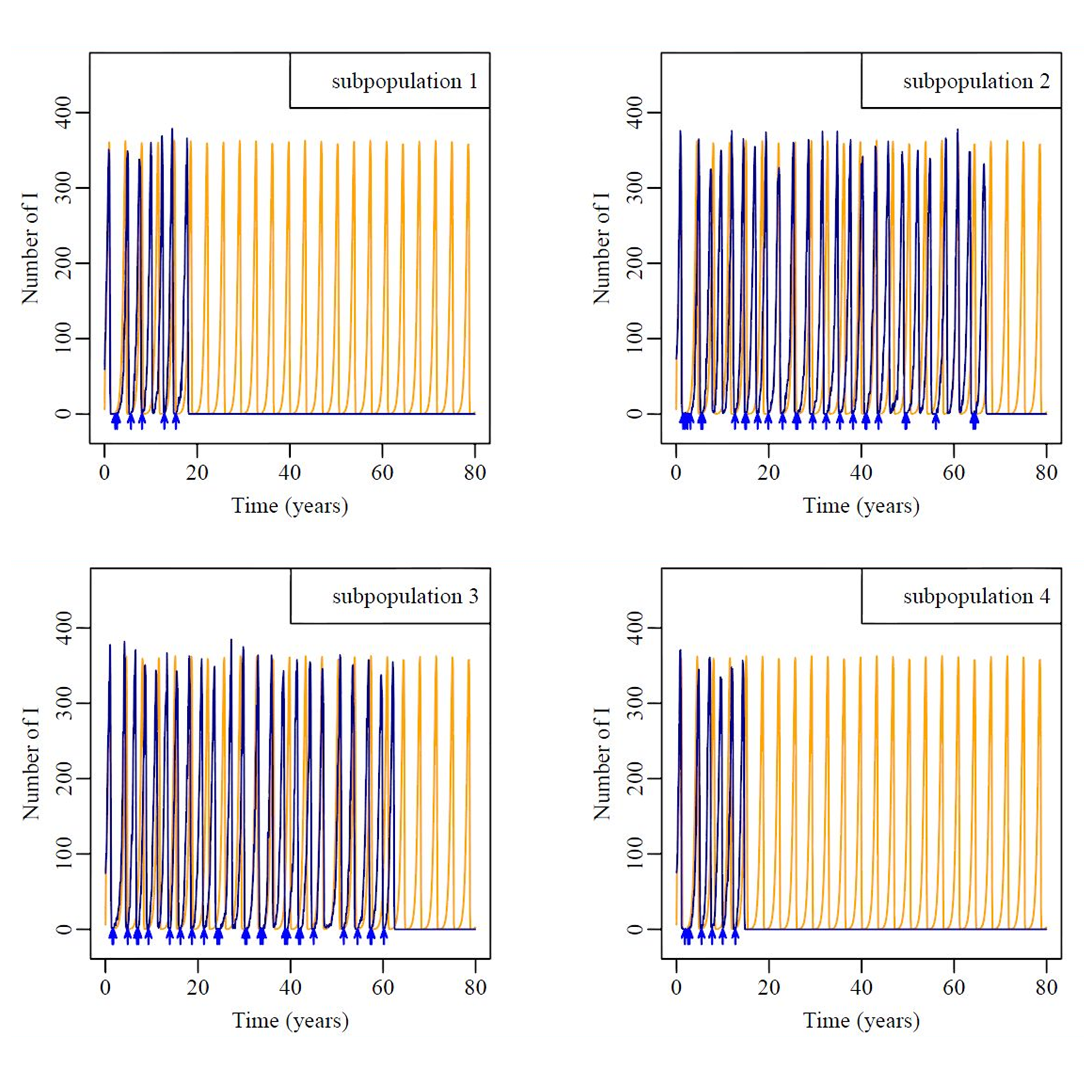

Supplement: Figure S10 — Extinction-recolonisation dynamics. Number of infectious rats through time in each subpopulation, for one of the stochastic simulations performed in Figure 4(b). In blue: stochastic results, in orange: deterministic results. , and other parameter values given in Table 1. The blue arrows indicate when the plague recolonizes a subpopulation from which it had disappeared (rescue effect). The disease never totally goes extinct in the deterministic model (epidemics still occur even if the number of infectious rats goes through extremely low values between the epidemics). (TIF) [file pcbi.1003039.s010.tif]

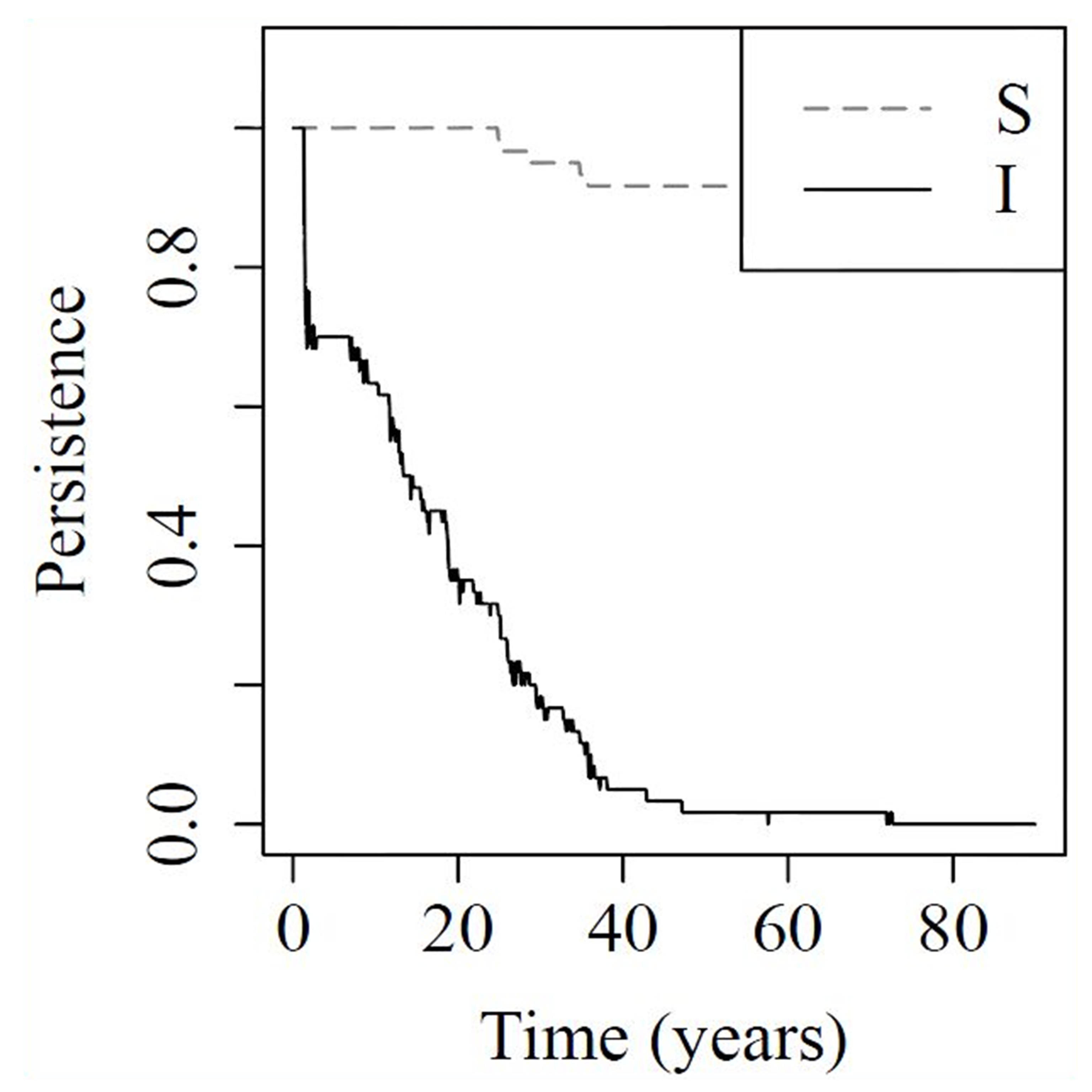

Supplement: Figure S11 — Effect of decreased force of coupling between subpopulations. Estimated probability of persistence of susceptible rats and infectious rats through time (measured on 60 simulations), for a metapopulation of 4 subpopulations with total rats and with proportion of inter-subpopulation infections (force of coupling between subpopulations) . , and other parameter values given in Table 1. This figure is to be compared with Figure 4(b). (TIF) [file pcbi.1003039.s011.tif]
