# Supplementary material for: Host Resistance, Population Structure and the Long-Term Persistence of Bubonic Plague: Contributions of a Modelling Approach in the Malagasy Focus
Source: PLoS Comput Biol. 2013 May 9;9(5):e1003039. doi: 10.1371/journal.pcbi.1003039 (PMC3649974; doi:10.1371/journal.pcbi.1003039)
Supplement: Text S1 — Other systems used. (PDF) [file pcbi.1003039.s012.pdf]

Text S1. Other systems used.

1. System (S1.1): transmission by vectors in a host population without resistant rats

$$\begin{aligned}
\frac{dS}{dt} &= rS\left(1-\frac{S+I}{K}\right) - dS - \frac{\beta FS}{S+I}(1-e^{-a(S+I)}) \\
\frac{dI}{dt} &= \frac{\beta FS}{S+I}(1-e^{-a(S+I)}) - (d+m)I \\
\frac{dN}{dt} &= r_f N\left(1-\frac{N}{K_f}\right) + \frac{F}{S+I}(1-e^{-a(S+I)}) \\
\frac{dF}{dt} &= (d+m)IN - d_f F - F(1-e^{-a(S+I)})
\end{aligned} \tag{S1.1}$$

2. System (S1.2): direct transmission in a host population without resistant rats

$$\begin{aligned}
\frac{dS}{dt} &= rS\left(1-\frac{S+I}{K}\right) - dS - \frac{\beta IS}{S+I} \\
\frac{dI}{dt} &= \frac{\beta SI}{S+I} - (d+m)I
\end{aligned} \tag{S1.2}$$

Infections are here realised by contacts between susceptible and infectious rats, so disease propagation is favoured by high transmission rates and limited by the mortality of infectious rats. The resulting equilibrium states are shown on the Supporting Figure S6. The threshold $R_0 = 1$ is reached for $\beta_0 = d+m$. The parameter K is not involved in this expression because transmission is supposed to be frequency-dependent [1].

References

1. McCallum H, Barlow N, Hone J (2001) How should pathogen transmission be modelled? Trends Ecol Evol 16: 295–300.
