# Supplementary material for: Host Resistance, Population Structure and the Long-Term Persistence of Bubonic Plague: Contributions of a Modelling Approach in the Malagasy Focus
Source: PLoS Comput Biol. 2013 May 9;9(5):e1003039. doi: 10.1371/journal.pcbi.1003039 (PMC3649974; doi:10.1371/journal.pcbi.1003039)
Supplement: Text S2 — Calculation of the propagation threshold by the Next Generation Approach. (PDF) [file pcbi.1003039.s013.pdf]

Text S2. Calculation of the propagation threshold by the Next Generation Approach.

The Next Generation Approach [1] is used to calculate the basic reproductive number of the disease, R_0. This method defines R_0 as the dominant eigenvalue of the Next Generation Operator. The Jacobian matrix of the variables transmitting the disease is evaluated at the disease extinction point and written as $J = M - D$, where M corresponds to the events where infectious individuals appear and D to the events where infectious individuals disappear ($M \geq 0$ and D a diagonal matrix > 0). The dominant eigenvalue of the matrix MD^{-1} corresponds to the number R_0.

The individuals which carry the disease are the infectious rats I and the free infectious fleas F. We consider $\frac{dI}{dt} = g$ and $\frac{dF}{dt} = f$. The Jacobian matrix we study is:

$$J = \begin{pmatrix} \frac{dg}{dI} & \frac{dg}{dF} \\ \frac{df}{dI} & \frac{df}{dF} \end{pmatrix} = \begin{pmatrix} -(d+m) & \beta \frac{S}{S+I+R}(1-e^{-a(S+I+R)}) \\ N(d+m) & -d_f - (1-e^{-a(S+I+R)}) \end{pmatrix} \tag{1}$$

The Jacobian matrix evaluated in ($S^* = K$, $I^* = 0$, $R^* = 0$, $N^* = K_f$, $F^* = 0$) is:

$$J^* = \begin{pmatrix} -(d+m) & \beta(1-e^{-aK}) \\ K_f(d+m) & -d_f - (1-e^{-aK}) \end{pmatrix} \tag{2}$$

$$= \begin{pmatrix} 0 & \beta(1-e^{-aK}) \\ K_f(d+m) & 0 \end{pmatrix} - \begin{pmatrix} (d+m) & 0 \\ 0 & d_f + (1-e^{-aK}) \end{pmatrix} \tag{3}$$

J^* can thus be written as (M - D).

$$MD^{-1} = \begin{pmatrix} 0 & \frac{\beta(1-e^{-aK})}{d_f+(1-e^{-aK})} \\ K_f & 0 \end{pmatrix} \tag{4}$$

The eigenvalues of MD^{-1} are $\pm\sqrt{\frac{\beta K_f}{1+\frac{d_f}{1-e^{-aK}}}}$.

Therefore,

$$\rho(MD^{-1}) = R_0 = \sqrt{\frac{\beta K_f}{1 + \frac{d_f}{1-e^{-aK}}}} \tag{5}$$

.

References

1. Diekmann O, Heesterbeek J (2000) Mathematical epidemiology of infectious diseases: model building, analysis and interpretation. Chichester: John Wiley.
